# *Mycoplasma bovis* infection alters small extracellular vesicle cargo derived from bovine endometrial epithelial cells cultured in static bioreactors

**DOI:** 10.3389/fmicb.2026.1770401

**Published:** 2026-05-07

**Authors:** Joel T. Pratt, Alice V. R. Lake, Evelyne Maes, Ancy Thomas, Charles Hefer, Murray D. Mitchell, Axel Heiser, Mallory A. Ross

**Affiliations:** 1 Bioeconomy Science Institute, Hopkirk Research Institute, AgResearch Group, Palmerston North, New Zealand; 2 Bioeconomy Science Institute, AgResearch Group, Lincoln, New Zealand; 3 Institute of Health and Biomedical Innovation, Queensland University of Technology, Brisbane, QLD, Australia

**Keywords:** biomarker, bioreactor, diagnostics, exosomes, *Mycoplasma* *bovis*, proteomics, small extracellular vesicles

## Abstract

*Mycoplasma bovis* (*M. bovis*) is a pathogenic bacterium that causes significant production losses and welfare challenges in cattle. Eradication is challenging as *M. bovis* can infect host cells intracellularly, reducing detection from immunomodulatory cells. With an aim to improve diagnostics for post-eradication surveillance, small extracellular vesicles (sEVs) were used to identify novel biomarkers of *M. bovis* infection. Readily accessible in body fluids, these nanoparticles encapsulate protein cargo indicative of the metabolic state of their tissue of origin. Here, we developed a novel three-dimensional *in vitro* cell culture model using static bioreactors to investigate changes in host cell (bovine endometrial epithelial cells) sEV protein cargo during *M. bovis* infection. Three-dimensional cell culture modelling better mimics native tissue architecture than two-dimensional modelling, enhancing intercellular function and physiological relevance. Size exclusion chromatography columns were used to isolate sEVs from control and co-culture bioreactor flasks. Liquid chromatography-trapped ion mobility spectrometry-tandem mass spectrometry was used to investigate the proteome of sEVs. Differential proteome profile analysis resulted in 193 proteins that demonstrated significantly different abundance (*p* < 0.05) in sEVs (co-culture vs. control cells), with changes to host Histone proteins the most prominent. Most *M. bovis*-derived proteins were those involved in cellular metabolism with enrichment of proteins classified under glycolysis. However, owing to their reduced genome, several glycolytic enzymes in *Mycoplasma* spp. are multifunctional and contribute to pathogenesis through involvement in adhesion and invasion of host cells. Homologs of immunoreactive *M. bovis-*lipoproteins were present in the proteome of co-culture sEVs. Host-derived proteins were consistent with those linked with infection and sEV trafficking. Our study indicated that sEV protein cargo was altered by *M. bovis* infection, with results providing an important insight into sEV biology in the context of bacterial infection.

## 1 Introduction

*Mycoplasma bovis* (*M. bovis*), a parasitic bacterium, is one of the major pathogens affecting global beef and dairy industries. *M. bovis* infections lead to severe welfare and economic challenges as all ages and both sexes of cattle can be affected (Maunsell et al., 2011; Dudek et al., 2020). Clinical signs manifest as endometritis, mastitis, arthritis, and bronchopneumonia (Calcutt et al., 2018). Treating *M. bovis* infection is challenging as resistance to common antimicrobials is increasing and although a commercial vaccine is available (Zoetis Inc), its effectiveness remains to be seen (Ledger et al., 2020; Dudek et al., 2021). Transmission of *M. bovis* in calves can occur through consumption of contaminated colostrum or milk, with *M. bovis* persisting for weeks after clinical signs resolve (Arcangioli et al., 2012). Additionally, semen is a source of infection with breeding bulls and artificial insemination contributing to transmission (Haapala et al., 2018).

Diagnostic testing is used to identify asymptomatic carriers that perpetuate herd reinfection (Petersen et al., 2020). Commercially available diagnostic tests, including enzyme-linked immunosorbent assay (ELISAs), polymerase chain reaction (PCR), and bacterial culturing, all have their limitations. Diagnostic testing is reliant on the extent that *M. bovis* is shedding at the time of sampling (Hazelton et al., 2018). Indirect ELISAs depend on seroconversion to detect infection, which can take weeks following initial exposure (Wawegama et al., 2014; Parker et al., 2017). Real-time PCR of tissue swabs and bulk milk samples demonstrate varied success as genetic diversity between strains can lead to false negatives (Behera et al., 2018; Register et al., 2018). Additionally, identification of sub-clinical animals is constrained by PCR sensitivity (Hazelton et al., 2020). Culturing *M. bovis in vitro* is time-intensive, requires specific conditions and, without sequencing, lacks specificity to distinguish *M. bovis* from closely related *Mycoplasma* spp. (Justice-Allen et al., 2011). Bulk milk testing can result in false negatives due to other bacteria outgrowing *M. bovis* during culturing (Parker et al., 2016). Discovery of a biomarker that reliably indicates *M. bovis* infection across all clinical conditions could reduce impacts on global welfare by strengthening disease detection and complementing established diagnostic approaches. With the aim of identifying candidate host-derived biomarkers for future diagnostic development, we investigated infection-associated changes in host molecular signatures during *M. bovis* infection.

Small extracellular vesicles (sEVs) are membrane-bound nanoparticles formed through two distinct mechanisms: (i) exosomes (30–150 nm), released via fusion of multivesicular bodies with the plasma membrane through endosomal sorting complex required for transport (ESCRT)-dependent pathways, and (ii) smaller ectosomes, formed through direct plasma membrane budding (Théry et al., 2018). sEVs are promising candidates for liquid biopsy, as their proteome, lipid composition, and nucleic acid content contain a variety of biomarkers representative of the metabolic and pathological state of their cell of origin (Alix-Panabières et al., 2023). Beyond being a by-product of cellular homeostasis, sEVs function as active mediators of intercellular communication by shuttling cargo (including proteins, lipids, metabolites, and nucleic acids) between adjacent and distant cells (Yáñez-Mó et al., 2015; Dixson et al., 2023). Comparing differences in the sEV cargo of patients with cancer (Hosseini-Beheshti et al., 2012; Sheridan, 2016), renal disease (Hogan et al., 2015), liver disease (Povero et al., 2020), Alzheimer’s disease (Kapogiannis et al., 2015), or active *Mycobacterium tuberculosis* infection (Kruh-Garcia et al., 2014), has aided diagnostic testing for these life-threatening illnesses. Critically, host sEV biogenesis and cargo composition are manipulated by tumours and intracellular pathogens to mask their pathological state and promote tolerance in naïve cells (Raab-Traub and Dittmer, 2017; Rabe et al., 2021). Changes in host sEV biogenesis is pathogen specific, for example: Human Cytomegalovirus infection of fibroblast cells upregulates host endosomal sorting proteins, causing a reduction in size of sEVs but an increase in sEV concentration (Streck et al., 2020); whereas, in patients infected with Human Immunodeficiency Virus-1, sEV size was larger than uninfected patients, though sEV abundance was increased (Hubert et al., 2015). Owing to a reduced genome, intracellular *M. bovis* is reliant on its host for survival and is likely to manipulate sEV biogenesis through presence of virulence factors (lipoproteins, glycolytic enzymes, adhesins, and nucleases) (Nunoya et al., 2020). Additionally, *Mycoplasma* spp. release sEVs with some containing toxins that can cause host cell apoptosis (Sreenath et al., 2026). Recent evidence demonstrated that *M. bovis* and *Mycoplasma gallisepticum* release sEV, which manipulated the immune function of their eukaryotic host cell (Wang et al., 2024; Wagner et al., 2025). If *M. bovis* manipulates host cells to tolerate its persistence, analysing changes in host sEVs secreted by *M. bovis* infected cells may provide novel disease biomarkers that could improve diagnostic testing for *M. bovis*.

Traditional two-dimensional (2D) *in vitro* co-cultures of *M. bovis* and various cell types have been established successfully (Bürki et al., 2015b; Josi et al., 2018). However, traditional 2D cell culture has limitations. Cell monolayers change their morphology and metabolism when grown in 2D, which affects cell to cell interactions, gene expression and cell proliferation (Chen et al., 2012; Costa et al., 2016; Langhans, 2018). Replicating the *in vivo* complexity of *M. bovis* infection requires use of a three-dimensional (3D) static bioreactor culture system, thereby creating conditions and cell interactions that enhance mimicry of physiological conditions (Souza et al., 2018). 3D cell culture techniques are designed to replicate *in vivo* complexity of intercellular communication and extracellular vesicle secretion (Koledova, 2024). Furthermore, as sEV production is limited by 2D cell culture (Patel et al., 2017), 3D culture conditions generate sufficient sEVs for downstream proteomic analyses.

In this work, our aim was to develop an *in vitro* model of bovine cells infected by *M. bovis*, using 3D co-culture to decipher changes in the sEV proteome as compared to an uninfected control cell culture. An advanced 3D culture model addresses key limitations of earlier systems, thereby enhancing relevance of sEV cargo profiling for biomarker discovery in *M. bovis* infection as conditions regarding cell morphology, metabolic activity and sEV yield are improved.

## 2 Materials and methods

### 2.1 Culturing

#### 2.1.1 Culturing and adapting a bovine endometrial epithelial cell line (bEEL cells)

**FIGURE 1:**
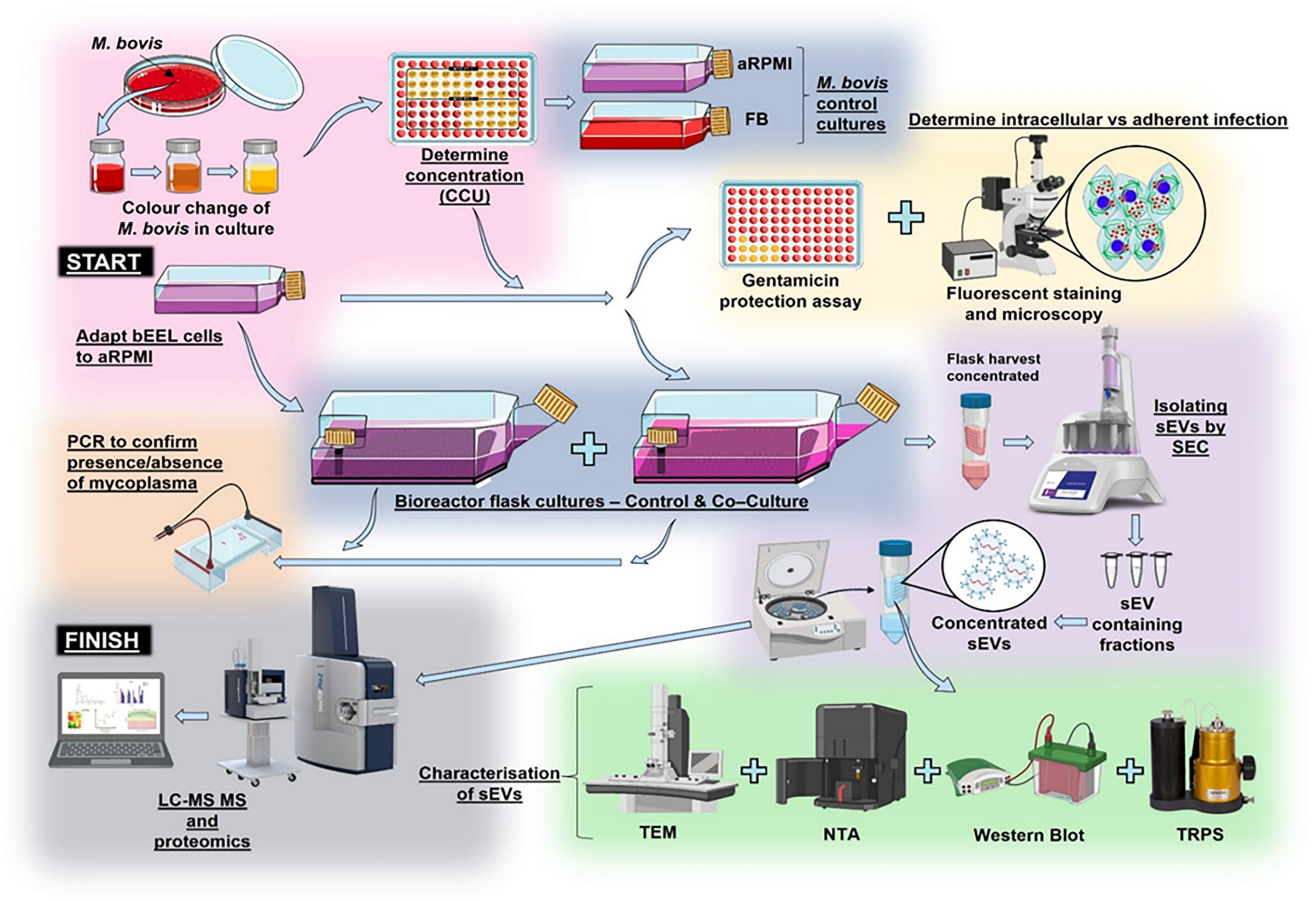
Experimental overview of an *in vitro* infection model with processing and characterisation techniques of small extracellular vesicles (sEVs). At the START point, two culturing methods proceeded concurrently. Mycoplasma bovis (*M. bovis*) was cultured in Friis broth (FB). Colour change of FB indicated *M. bovis* concentration using colour change units (CCU). A bovine endometrial epithelial cell line (bEEL cells) was adapted to Advanced Rosewell Park Memorial Institute medium (aRPMI). Two CELLine AD1000 bioreactor flasks were seeded with adapted bEEL cells; one used as a control and the other as a co-culture of bEEL cells and *M. bovis*. In addition, two *M. bovis* controls were created: *M. bovis* grown in FB and *M. bovis* grown in aRPMI. Epi-fluorescent microscopy and a gentamicin protection assay assessed capability of *M. bovis* to infect bEEL cells. Polymerase chain reaction (PCR) assessed presence/absence of *M. bovis* in both bioreactor flasks. Using size exclusion chromatography (SEC), small extracellular vesicles (sEVs) were isolated, eluted into fractions and concentrated. Tunable resistive pulse sensing (TRPS), western blotting, and transmission electron microscopy (TEM) characterised sEVs. At the FINISH point, all sEVs were processed using liquid chromatography trapped ion mobility spectrometry tandem mass spectrometry (LC–TIMS-MS/MS) for proteomic analysis of sEV proteins assessed differences between the control and treatment groups (denoted by a grey background). Created using Servier Medical Art, licensed under a Creative Commons Attribution 3.0 Unported License (https://smart.servier.com/), and BioRender (https://biorender.com/).

Figure 1 initially, bEEL cells (Krishnaswamy et al., 2009) were cultured at 37 °C in a humidified 5% CO_2_ incubator (Heracell™ 150i CO_2_ incubator, ThermoFisher Scientific) in 15 mL of Rosewell Park Memorial Institute medium (RPMI) (Gibco, ThermoFisher Scientific) supplemented with 10% heat inactivated foetal bovine serum (FBS) (Gibco, ThermoFisher Scientific) and 1x Penicillin/Streptomycin (Gibco, ThermoFisher Scientific). Cells were passaged at ∼70–80% confluency, washed with phosphate buffered saline (1x PBS) and dissociated using TrypLE^®^ (Gibco, ThermoFisher Scientific) (Phelan and May, 2015). In a Nunc EasYFlask 75 cm^2^ (T75 flask) (Nunc, ThermoFisher Scientific), cells were resuspended in 15 mL of RPMI to achieve ∼30% confluency. Cells were imaged with an EVOS™ XL Core imaging system (Life Technologies, ThermoFisher Scientific). A TC20 automated cell counter (BioRad) and 0.4% trypan blue solution (Gibco, ThermoFisher Scientific) were used for cell counts, with live cells averaged from two cell counts (gated between 8 and 30 μm). This cell line was kindly donated by Michel A. Fortier (Universite Laval, Quebec, Canada).

Over 8 weeks, bEEL cells were adapted to grow in 15 mL of fresh advanced Rosewell Park Memorial Institute medium (aRPMI) (Gibco, ThermoFisher Scientific) (supplemented with 4.5% of exosome–depleted FBS (Gibco, ThermoFisher Scientific), 2x GlutaMAX supplement (Gibco, ThermoFisher Scientific) and 1x Penicillin/Streptomycin). Adaptation required an initial supplement change from 10% FBS to 10% exosome-depleted FBS. Fifteen passages were required to gently reduce the FBS requirement of bEEL cells to 4.5% from 10%. Three consecutive passages in aRPMI supplemented with 4.5% of exosome–depleted FBS, 2x GlutaMAX supplement and 1x Penicillin/Streptomycin confirmed bEEL cells were successfully adapted to their medium.

#### 2.1.2 Culturing *Mycoplasma bovis* (*M. bovis*)

A New Zealand strain (isolate W18_04866 (#1) of *M. bovis* was cultured in a physical containment level 3 (PC3) facility with approval under the Hazardous Substances and New Organisms Act 1996 (Crookenden, 2020).

*Mycoplasma bovis* was grown on Friis agar (FA) and cultured in 4 mL Friis broth (FB) in a humidified incubator at 37 °C/5% CO_2_. Light absorbance spectrophotometry (Ultrospec 2000 UV/Visible Spectrophotometer, Pharmacia Biotech), at wavelengths of 415 nm and 560 nm, was used to determine viability of FB aliquots by comparing colour change of a positive control (*Mycoplasma mycoides* subsp. *capri)*, a negative control (*Acholeplasma laidlawii*) and an uninoculated FB control over 9 days (Held, 2018). Subculturing *M. bovis* required a 1:400 dilution in fresh FB. Stocks of *M. bovis* were stored at −80 °C on cryobeads.

#### 2.1.3 Bioreactor flasks and T75 flasks for technical controls of *M. bovis* growth conditions

Two CELLine AD1000 bioreactor flasks (Merck–Millipore) were maintained for 3 months at 37 °C in a humidified 5% CO_2_ incubator. The cell compartment of each bioreactor flask (bEEL only and co-culture of *M. bovis* and bEEL cells) was seeded with adapted bEEL cells (29.25 × 10^6^ cells/15 mL of aRPMI). Once seeded, the bioreactor flasks were maintained for 1 month to ensure culture stability. The co-culture flask was inoculated once with *M. bovis* at a Multiplicity of infection (MOI) of 5 (1) (Poveda and Nicholas, 1998), which was not repeated, where: CCU = Colour Change Units (*M. bovis* concentration). A colour change assay (Ackerman et al., 2019) determined the concentration of a 4–day old subculture of *M. bovis* to be ∼10^9^ CCU/mL. Once *M. bovis* was inoculated, the co-culture bioreactor flask was not disturbed/harvested for a week to ensure infection stability.


M′⁢O⁢I′= (1)

$$\frac{M.b\,o\,v\,i\,s\,v\,o\,l\,u\,m\,e\,\left(\mu \,L\right)\times M.b\,o\,v\,i\,s\,c\,o\,n\,c\,e\,n\,t\,r\,a\,t\,i\,o\,n\,\left(C\,C\,U\right)}{C\,o\,n\,c\,e\,n\,t\,r\,a\,t\,i\,o\,n\,s\,o\,f\,b\,E\,E\,L\,c\,e\,l\,l\,s\,\left(c\,e\,l\,l\,s/m\,L\right)}$$

Separately, two T75 flasks were used as technical controls to monitor *M. bovis* growth conditions (without presence of eukaryotic cells). At 2.5% (v/v), *M. bovis* was inoculated into 15 mL of aRPMI or FB. These controls were maintained at 37 °C in a humidified 5% CO_2_ incubator.

### 2.2 sEV production

#### 2.2.1 Harvesting and processing sEVs

Once weekly, the cell compartment of each bioreactor was harvested (two biological samples). First, the media compartment of the bioreactor was emptied. The media compartment contained 1 L of supplemented aRPMI without 4.5% exosome-depleted FBS (serum-free aRPMI). Next, the bEEL cells adhered to the polyethylene terephthalate matrix within the cell compartment were washed gently three times with the 15 mL of sEV-rich aRPMI present in the cell compartment. The sEV-rich aRPMI was transferred into a sterile 15 mL conical tube. To feed the ongoing culture, 50 mL of fresh serum-free aRPMI was added into the media compartment, and the cell compartment was refilled with 15 mL of fresh aRPMI. Bubbles were removed to prevent matrix rupture. Finally, the media compartment was replenished with 1 L of fresh serum-free aRPMI. Technical controls of *M. bovis* growth conditions (1.1.3) were harvested concurrently.

#### 2.2.2 Clarifying and concentrating sEV harvests

To remove cell debris, harvested aRPMI was clarified at 500 ×*g*, then 10,000 × *g*, for 10 min. Before use, Amicon Ultra–15 centrifugal filter units (Ultracel–100 membrane) (CF units) (Merck–Millipore) were primed as described in manufacturer’s instructions. Using primed CF units, a proportion (6 mL) of clarified supernatant was concentrated at 4,000 × *g* until the retentate was ∼1 mL. The retentate was added to the remaining 9 mL of clarified supernatant, forming a final volume of 10 mL. Clarified sEV harvests (two biological samples, each with five technical samples) were frozen at −20 °C until sEV isolation occurred.

#### 2.2.3 Collecting and concentrating sEV fractions

Size exclusion chromatography (SEC) qEV10 columns (Izon Science, New Zealand) with 35 nm pores, and an Automatic Fraction Collector (AFC) (Izon Science, New Zealand), enabled isolation of sEVs sized between 30 and 150 nm (Yang et al., 2020). Prior to use, qEV10 columns were primed according to manufacturer’s instructions. Through gravity flow, all 10 mL of clarified supernatant passed through the column frit. The volume of degassed 1x PBS (sonic bath for 5 min at 94% power/75 Hz) added was determined by the AFC, which depended on the number of collected fractions required (variable, predetermined at the beginning of each run). Each 5 mL fraction was collected in Protein LoBind tubes (5.0 mL; PCR clean (Eppendorf AG), with all fractions stored at 4 °C until concentrated.

From each bioreactor harvest, the three sEV-containing fractions (5–7) were concentrated using primed CF units (see 1.2.2). CF units were spun at 4,084 × *g*, until the retentate was ∼500 μL. Each retentate was filtered using a sterile 0.1 μm Millex™ PVDF syringe filter (Sigma–Aldrich, Merck–Millipore). Retentates were filtered into Protein LoBind tubes (1.5 mL; PCR clean (Eppendorf AG) for downstream analyses.

qEV10 column quality control included assessing each eluted fraction using light absorbance spectrophotometry at wavelengths of 350 and 600 nm. Column blockages were determined by an averaged drop-rate/minute. Columns were reused up to five times, with reuse/storage of columns undertaken according to manufacturer’s instructions. Between uses, columns were stored at 4 °C.

#### 2.2.4 Fixation/Lysis of sEVs

From each sample (two biological samples, each with five technical samples), 10% of the total retentate volume was aliquoted for sEV analysis. From this 10% aliquot, 6 μL was fixed with 3% glutaraldehyde (1:1) for transmission electron microscopy (TEM).

The remaining volume of retentate was mixed (2:1) with lysis buffer: 7 M urea (Avantor, VWR International), 2 M thiourea (Avantor, VWR International), 1% sodium deoxycholate in 100 mM ammonium bicarbonate (Sigma–Aldrich, Merck–Millipore) dissolved in LC–MS/MS–grade water.

##### 2.2.4.1 Biosafety considerations

To ensure safe decontamination and removal from PC3 containment, in accordance with New Zealand biosecurity regulations for notifiable pathogens, all samples (lysed and viable) were heated at 95 °C for 10 min. All lysed and heat treated sEV samples were stored at −80 °C. Inactivation of *M. bovis* was confirmed by inoculating lysed samples into FB and by plating on FA. An absence of FB colour change and FA colony growth confirmed *M bovis* inactivation.

### 2.3 Assessing contamination

To ensure the absence or presence of *Mycoplasma* spp. in control and co-culture harvests, DNA was purified from clarified supernatant (1.2.2) using a DNeasy kit (Qiagen). A LookOut *Mycoplasma* PCR detection kit (Sigma–Aldrich, Merck–Millipore) was used to assess for nineteen *Mycoplasma* spp. PCR products were confirmed by gel electrophoresis through a 1.5% agarose gel for 40 min at 140 V. DNA was visualised with 1x RedSafe™ nucleic acid staining solution (iNtRON Biotechnology) and a ChemiDoc MP imaging system (Bio-Rad) using system set ethidium bromide settings. A GeneRuler™ 50 bp DNA ladder (ThermoFisher Scientific) was used for size reference of PCR product.

### 2.4 Analysis of sEVs

#### 2.4.1 Transmission electron microscopy (TEM)

Each fixed control and co-culture sEV sample was diluted 1:5 with LC–MS/MS–grade water. Formvar/carbon-coated size-200 mesh copper grids (2 mm) (Sigma–Aldrich, Merck–Millipore) were inverted into 10 μL of each sample for 4 min, washed once with LC–MS/MS–grade water and negatively stained for 4 min using 10 μL of an aqueous 4% (w/v) uranyl acetate solution. Images were captured using a Tecnai G2 Spirit BioTWIN TEM (Field Electron and Ion Company, ThermoFisher Scientific) at magnifications of 43,000x and 135,000x. To establish a size distribution graph, five images at the same magnification with a minimum of fifty sEVs were measured manually using the measurement tool in ImageJ (Fiji) software.

#### 2.4.2 Nanoparticle tracking analysis (NTA)

Concentrated sEV from *M. bovis* negative samples were diluted 1:1,000 in 1 × PBS for NTA using a NanoSight NS300 instrument (Malvern Panalytical, United Kingdom). Running settings included a camera level of 13 and pump speed of 300. Six videos per sample were captured for analysis with a modal size and concentration of sEVs across all three videos reported. Analysis settings included a detection threshold of 10. Water (1 mL; Milli-Q, Merck-Millipore) was used to clean the line between samples and 2 × water (1 mL) and 1 × 5% ethanol (500 μL) was infused after each batch of samples on a given day.

#### 2.4.3 Tunable resistive pulse sensing (TRPS)

Using the qNANO (Izon Science), sEV size/concentration were measured. Blockages of a 100 nm polyurethane nanopore, NP100 (Izon Science) were interpreted as a current, which defined sEV size/concentration. The qNANO was initialised and calibrated according to manufacturer’s recommendations using 100 nm polystyrene calibration particles, CPC100 (Izon Science), diluted 1/1,000 in 1x PBS/0.05% (v/v) Tween–20. Pore stretching, voltage and applied pressure were adjusted to ensure a baseline current of > 100 nA and background noise (root mean square) of < circa 15 pA. Calibrations and measurements were evaluated using two positive pressures, 5 and 10 mbar. A minimum of 500 blockade events were required for each measurement. To ensure pore stability and no blockages had occurred, the qNANO was re-calibrated after each sample measurement. Data was analysed using the Izon Control Suite 3 software (Izon Science).

### 2.5 Analysis of sEV proteins—western blotting

To assess positioning of sEV protein markers, 120 μL from each fraction (2–11, isolated from control sEV harvests using the AFC) were mixed 1:1 with radioimmunoprecipitation assay buffer and agitated for 30 min at 4 °C. Samples were sonicated on ice (1,000 kJ, 10 s pulses totaling 1 min) and centrifuged at 12,000 × *g* for 20 min at 4 °C. Lysates were concentrated to ∼120μL using a CentriVap benchtop vacuum concentrator (Labconco, MO, United States) and stored at −80°C in 1.5mL Protein LoBind tubes. Lysates were normalised by mixing 10% of total volume with 4 μL NuPAGE™ LDS sample buffer (4×) (2.5% β-mercaptoethanol). Samples were heated at 90 °C for 5 min prior to loading into Bis-Tris gels (15-well, 1.5 mm, 4–12%, ThermoFisher Scientific) with 1 × MES/SDS buffer and NuPAGE antioxidant. A SeeBlue™ Plus2 pre-stained protein standard (4 μL) was used. Protein transfer occurred using an iBlot™ transfer stack and iBlot device (ThermoFisher).

Membranes were blocked for 1 h at room temperature in 5% skim milk (w/v) in 1 × TBS-T (0.1% Tween-20), then washed twice in TBS-T. Primary antibodies (1:1,000 in blocking buffer) targeting CD63, CD9, HSP70 (Exo-Ab-Kit-1, System Biosciences), Syntenin-1 (Ab19903, Abcam), and Calnexin (Ab22595, Abcam) were incubated overnight at 4°C with agitation. Membranes were washed five times with TBS-T, then incubated for 1 h at room temperature with anti-rabbit HRP-conjugated secondary antibody (1:10,000 in blocking buffer), followed by five TBS-T washes. Clarity™ ECL substrate (Bio-Rad) and the ChemiDoc MP system were used for imaging. Ladder and protein bands were processed using ImageLab software (Bio-Rad).

### 2.6 Gentamicin protection assay

A gentamicin protection assay was used to assess if *M. bovis* was limited to extracellular adherence of bEEL cells or if intracellular infection occurred (Raymond et al., 2018).

Bovine endometrial epithelial cells were seeded in sixteen wells of a 24-well flat–bottomed cell culture plate (ThermoFisher Scientific) at 2 × 10^5^ cells/well. Eight wells remained unseeded (*M. bovis*–only controls). Host bEEL cells were infected with *M. bovis* at a MOI of 5 (1). The plate was shaken at 300 rpm for 10 min, centrifuged at 300 × *g* for 5 min and transferred to a 37°C/5% CO_2_ humidified incubator for twenty-4 h. Once incubated, all wells were washed five times with 1 mL of 1x PBS. Gentamicin (10 mg/mL, Gibco, ThermoFisher Scientific) was diluted in aRPMI (to 200, 100, 50, and 25 μg/mL) and added to specific wells. Gentamicin treated cells were incubated for 3 h. Following incubation, gentamicin was removed, and all wells were washed five times with 1 mL of 1x PBS. Using TrypLE^®^ (20 μL/well), adherent cells were dissociated through incubation at 37°C for 5–10 min. To assess *M. bovis* protection, in duplicate, 20 μL of dissociated bEEL cells were mixed with 180 μL of FB in column 1 of a 96-well sterile, flat-bottomed cell culture plate (ThermoFisher Scientific). Cells were 10-fold serially diluted across the plate (column 1–12, 10^1^–10^11^ CCU). Plates were sealed with parafilm and incubated at 37 °C/5% CO_2_ for 2 weeks. The pH/colour change was compared to uninfected control cells using light absorbance spectrophotometry at wavelengths of 415 and 560 nm.

### 2.7 Epi–fluorescent microscopy

Round glass coverslips (22 mm) were sterilised in 70% acetone and 70% ethanol. Sterile coverslips were placed into each well of a 12–well cell culture plate (Nunc, ThermoFisher Scientific). Coverslips were seeded with bEEL cells at 2 × 10^5^ cells/mL (1.1.1) and incubated for twenty-4 h before infection (bEEL cells estimated to grow to 5 × 10^5^ cells/mL over two and a half days).

A 4–day old subculture of *M. bovis* isolate W18_04866 (#1) (1 × 10^9^ CCU/mL) was aliquoted to achieve a MOI of 10 (1) in 1 mL of FB. Separately, 1 mL FB-only negative-control aliquots were prepared. Both aliquot types were stained with octadecyl rhodamine B chloride (R18) at 5 μg/mL and incubated for 15 min in the dark at 37 °C in a humidified 5% CO_2_ incubator. After staining, both aliquot types were centrifuged at 12,000 × *g* for 15 min, the supernatant removed, and the pellets were washed with 1 mL of 1x PBS. They were then centrifuged twice at 12,000 × *g* for 30 min, with supernatant removal and pellet washing occurring between spins. Final pellets (*M. bovis* positive FB and FB-only negative control dye residue) were resuspended in 1 mL of supplemented aRPMI (1.1.1).

The aRPMI on coverslips containing adhered bEEL cells was removed, and cells were washed with 1 mL of 1x PBS. Then, 1 mL of the resuspended stained *M. bovis* preparation or dye residue control was added to the corresponding coverslip. Cell infection proceeded for twenty-4 h at 37 °C in a humidified 5% CO_2_ incubator. Post-infection, coverslips were washed three times with 1 mL of 1x PBS. Cells were fixed with 200 μL of 10% (v/v) neutral buffered formalin for 15 min, then washed with 1 mL of 1x PBS. Once fixed, cells were permeabilised for 5 min at room temperature with 0.05% (v/v) Triton X–100/1x PBS, then washed with 1 mL of 1x PBS.

Fixed cells were stained in 50 μL of their respective post–stain (fluorescein isothiocyanate (FITC)–Phalloidin (final concentration; 1 μg/mL), and 4′,6–diamidino–2–phenylindole (DAPI; 2 μg/mL) by inversion of coverslips onto parafilm in a dark humidified chamber for 30 min. Stains were prepared in the dark and diluted in 1x PBS. All coverslips were washed five times using 1 mL of 1x PBS. ProLong™ Gold antifade mountant (ThermoFisher) was used to mount coverslips onto microscope slides. Coverslips were imaged on a 20x long–working objective on an Eclipse Ti–U inverted epi–fluorescent microscope (Nikon) using Texas Red, FITC, and DAPI. All images were processed using ImageJ (Fiji) software.

### 2.8 Liquid chromatography trapped ion mobility spectrometry tandem mass spectrometry (LC–TIMS-MS/MS)

#### 2.8.1 Processing of sEV proteins

Thawed sEV lysates (two biological samples, each with five technical samples) were sonicated three times for 5 s using a VC–50 Vibra cell ultrasonic processor (Sonics & Materials Inc., Newtown, CT, United States) at an output of 30 W. Lysates were centrifuged at 12,000 × *g* for 10 min at 4 °C, with clear supernatants collected. Aliquots (100 μL) were precipitated using methanol/chloroform procedure as described in Wessel and Flügge (1984). Air-dried protein pellets were resuspended in 100 μL of ammonium bicarbonate (10 mM). Protein concentrations were determined using Qubit 1.0 fluorometer (Invitrogen, ThermoFisher Scientific) and a QuDye protein quantification kit (Lumiprobe).

#### 2.8.2 Digestion of proteins to peptides

An aliquot containing 50 μg of proteins was reduced by addition of 1 μL of 200 mM dithiothreitol, followed by incubation in darkness at 56 °C for forty–5 min in a thermomixer (Eppendorf AG). Proteins were alkylated by addition of 1 μL of 200 mM iodoacetamide in 0.1 M ammonium bicarbonate, followed by incubation in darkness at room temperature (∼20°C) for 30 min in a thermomixer. Proteins were then digested with 2 μg of MS–grade Pierce™ trypsin/Lys–C protease mix (ThermoFisher Scientific) (1:25 enzyme:protein) in the presence of 10% acetonitrile, followed by incubation overnight at 37 °C in a thermomixer. The resulting peptides were desalted with Pierce™ 100 μL C18 Tips (ThermoFisher Scientific), dried using a centrifuge concentrator and resuspended in 0.1% formic acid prior to LC–TIMS-MS/MS analysis.

#### 2.8.3 Data acquisition

Data acquisition by LC-TIMS-MS/MS was enabled using a nanoElute nanoflow ultra-high pressure LC system (Bruker Daltonics, Bremen, Germany) coupled to a timsTOF Pro2 mass spectrometer (Bruker Daltonics), which was equipped with a CaptiveSpray ion source (Bruker Daltonics). The mass spectrometer was operated in positive-ion mode. For each sample, 200 ng of peptides were loaded onto a reversed-phase column (25 cm length, 75 μm inner diameter, 1.6 μm particle size, 120 Å pore size) with a pulled-emitter tip (IonOpticks, VIC, Australia). Peptides were separated at 50 °C at a flow rate of 300 nL/min using a multi-step linear gradient of mobile phase A (0.1% formic acid in water) and mobile phase B (0.1% formic acid in 100% acetonitrile), increasing from 2 to 35% B in 60 min, then a steep increase to 95% B in 10 min. The column was held at 95% B for 5 min, returned to 2% B over 2 min, and then re-equilibrated at 2% B for 13 min (resulting in a total runtime of 90 min).

The timsTOF Pro 2 was operated in parallel accumulation–serial fragmentation (PASEF) data-dependent acquisition (DDA) mode using ten PASEF MS/MS scans per cycle. For proteomic analysis, a polygon filter was applied to exclude low m/z, singly charged ions from PASEF precursor selection. The TIMS-MS survey scan was acquired between 0.6 and 1.6 V s/cm2 and 100–1,700 m/z with a ramp time of 166 ms. The ten PASEF scans contained maximum of twelve MS/MS scans per PASEF scan with a collision energy of 10 eV. Precursors with 2–5 charges were selected with the target value set to 10,000 a. u. and intensity threshold to 2,500 a. u. Precursors were dynamically excluded for 0.4 s.

#### 2.8.4 Protein identification

The PEAKS Online software package (Bio informatic Solutions Inc.) was used to analyse the LC-TIMS-MS/MS data. Raw data were refined by a built-in algorithm that enabled association of chimeric spectra. Proteins/peptides were identified with the following parameters: precursor mass error tolerance of 20 ppm, fragment mass error tolerance of 0.05 Da, in-house database with *Bos taurus* and *M. bovis* (UNC_01) (v2020.06, 65878 sequences), *Saccharomyces cerevisiae* Uniprot database (v2022.01, 6638 sequences) as a contaminant database, trypsin/LysC specified as digestive enzyme and up to two missed cleavages were allowed. Carbamidomethylation of cysteine was set as fixed modification. Oxidation (M), carbamylation (C) and deamidation (NQ) are chosen as variable modifications. A maximum of three post-translational modifications per peptide were permitted. False discovery rate (FDR) estimation was made based on decoy-fusion. An FDR of 1% was set at peptide level and a −10–log p > 20 at protein level, with at least one unique peptide per protein considered adequate for confident protein identification.

#### 2.8.5 Label free quantification

Label-free quantification was performed using the quantitation node of Peaks Online software, enabling comparison of protein expression levels between the distinct groups. Alignment used a mass-tolerance error of 20 ppm, and a maximum retention-time shift tolerance of 2 min. Relative protein and peptide abundances in RT-aligned samples were determined by peptide-feature-based quantification. All samples were normalised using total ion chromatogram normalisation relative to a quality control sample, and identity transfer within groups was allowed. Relative comparison between samples is based on the area under the curve. Relative comparisons between samples were based on the cumulative area under the curve, calculated from unique peptides assigned to each protein, which were limited to the top 10 highest-quality peptides. The FDR was set at 1%.

#### 2.8.6 Statistical analysis

Unsupervised and supervised multivariate classification methods were used to identify proteins that contributed to the separation of samples into control and treatment groups. All data analyses were performed in the R statistical software package, using a range of software libraries (mixOmics, sva, tidyverse, ggplot2, PerformanceAnalytics, and factoextra). Differential abundance calculations between bEEL vs. CC were determined using an adjusted *p*-value cutoff of 0.05. Analyses regarding *M. bovis* recovery or inactivation were performed using either an unpaired students t–test or a One–Way ANOVA. The significance values were indicated as * = *p* < 0.05, ** = *p* < 0.01, *** = *p* < 0.001, **** = *p* < 0.00001, or ns = not significant. Analyses of *M. bovis* growth were performed using a repeated measures ANOVA.

Principle component analysis (PCA) was performed on protein abundance levels using the PCA function of the mixOmics package (Rohart et al., 2017). Prior to calculating PCA, proteins were filtered to only include those with an abundance estimate of at least 25% within each group. Protein differential abundance analyses were calculated by fitting a negative binomial model (glm.nb from the R package MASS (R Core Team, 2013; Venables and Ripley, 2013) to estimate the abundance as an interaction between the protein and sample group, followed by the adjusted p–value calculation for each individual protein in each group using the R package predictmeans (version 1.0.6, ((Dongwen, 2021)).

#### 2.8.7 Data visualisation

Data visualisation was performed using R (version 3.6.1) using the tidyverse set of data transformation tools and the ggplot package to construct graphs. Pathway analysis was performed using PANTHER. The exocarta database was used to compare protein identifications with known exosomal proteins.

#### 2.8.8 PRoteomics identifications database (PRIDE)

The mass spectrometry proteomics data have been deposited to the ProteomeXchange Consortium via the PRIDE [1] partner repository with the dataset identifier PXD076118 and 10.6019/PXD076118.

## 3 Results

### 3.1 Presence of *M. bovis* in the co–culture bioreactor was consistent throughout culturing

**FIGURE 2:**
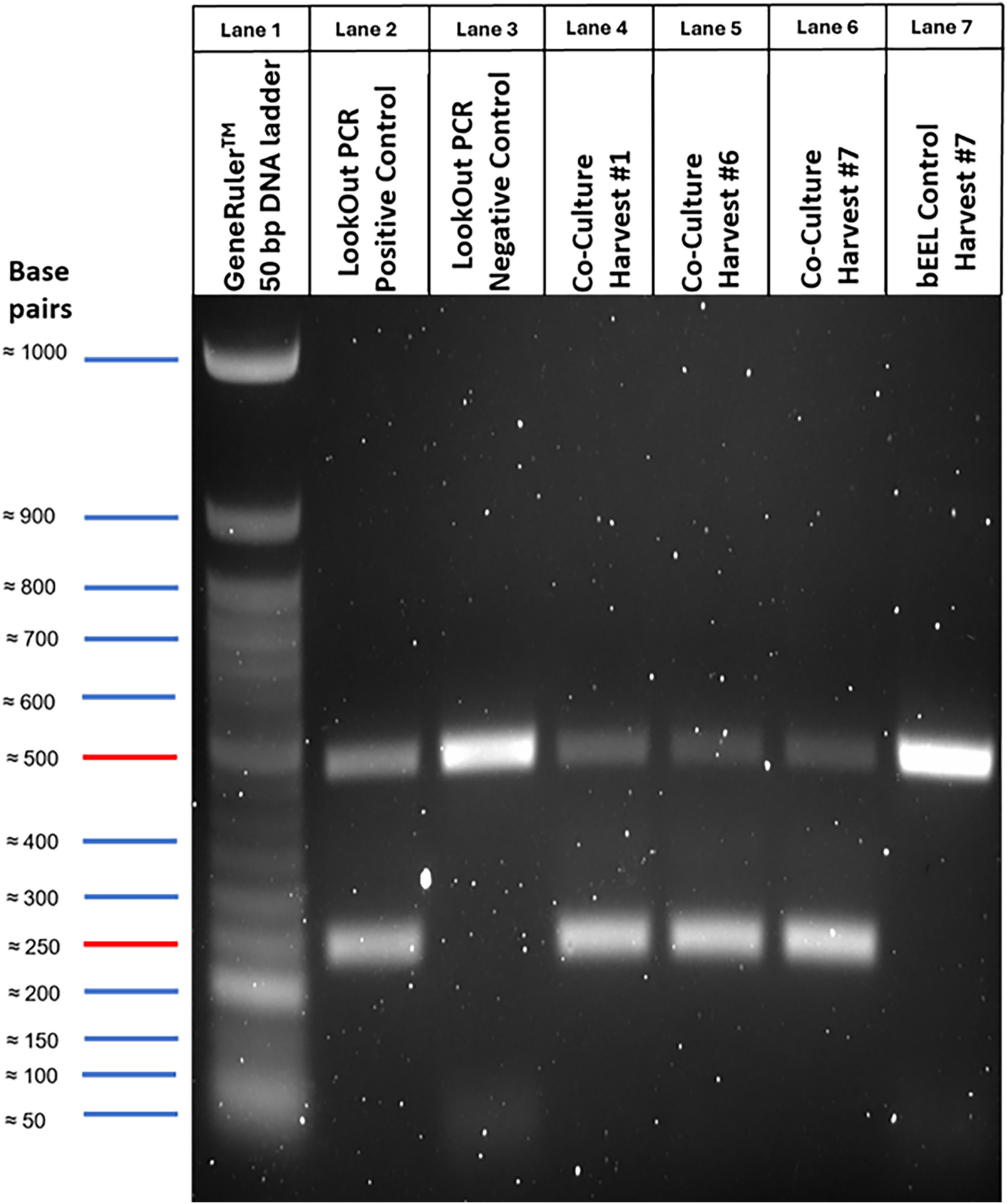
Presence and/or absence of *Mycoplasma bovis* (*M. bovis*) isolate W18_04866 (#1) in bioreactor flasks was confirmed through Polymerase Chain Reaction (PCR) of harvested cell culture media. A positive *Mycoplasma* spp. (lane 2) control and negative (lane 3) control provided by the LookOut Mycoplasma PCR Detection Kit (Sigma–Aldrich, Merck–Millipore) were included. Lanes 4–6 represent co-culture bioreactor harvests (Harvest 1, 6, and 7, respectively; which represents weeks 1, 6, and 7 post-inoculation of *M. bovis* into the bioreactor flask). Lane 7 represents a harvest of the bovine endometrial epithelial cells (bEEL cells)-only control bioreactor (taken 3 months post initial seeding of bEEL cells). Harvests 2–5 were collected weekly but were not included, as the study focused on demonstrating infection establishment and persistence rather than temporal progression. A GeneRuler™ 50 bp DNA ladder (ThermoFisher Scientific) (lane 1) was used. A band at ∼500 bp confirmed viability of the kit; a second band at ∼250 bp confirmed presence of a *Mycoplasma* sp.

PCR confirmed the presence of a *Mycoplasma* spp. in the co-culture bioreactor and its absence in the bEEL-only control bioreactor (Figure 2). The bioreactor flasks were maintained for 3 months total: 1 month for establishment and stabilisation of bEEL cells prior to *M. bovis* inoculation, followed by 2 months of co-culture. Weekly harvests were performed throughout the 2-month co-culture period (Harvests 1–7). For gel analysis, three timepoints were selected to demonstrate *M. bovis* persistence: Harvest 1 (1-week post-inoculation, which confirmed initial establishment of *M. bovis* infection), and Harvests 6 and 7 (6 and 7-weeks post-inoculation, which confirmed sustained infection). Harvests 2–5 were not displayed as the primary objective was to demonstrate consistency of *M. bovis* presence at the beginning and end of the infection period. Co-culture harvest samples (lanes 4, 5, and 6, representing Harvests 1, 6, and 7 respectively) were comparable to an included *Mycoplasma* spp. positive control (lane 2), confirming stable *M. bovis* colonisation. The bEEL-only control bioreactor harvest (lane 7, sampled at 3 months post bEEL cell establishment) was comparable to an included negative *Mycoplasma* spp. control (lane 3), confirming all nineteen *Mycoplasma* spp. (including *M. bovis*) were completely absent in the control bioreactor. Since *M. bovis* was specifically inoculated into the co-culture bioreactor flask, the *Mycoplasma sp.* detected in lanes 4, 5, and 6 was confirmed as *M. bovis* isolate W18_04866 (#1).

### 3.2 *Mycoplasma bovis* was protected by bEEL cell presence in a gentamicin protection assay

At all concentrations of gentamicin tested (200–25 μg/mL), *M. bovis* only controls were unable to cause a colour change of FB after 2 weeks of growth. Eukaryotic bEEL cells provided protection for intracellular *M. bovis* against gentamicin concentrations of 25, 50, and 100 μg/mL. However, at 200 μg/mL, there was no colour change of FB after 2 weeks growth.

### 3.3 Pre–stained *M. bovis* was present in post–infected bEEL cells

**FIGURE 3:**
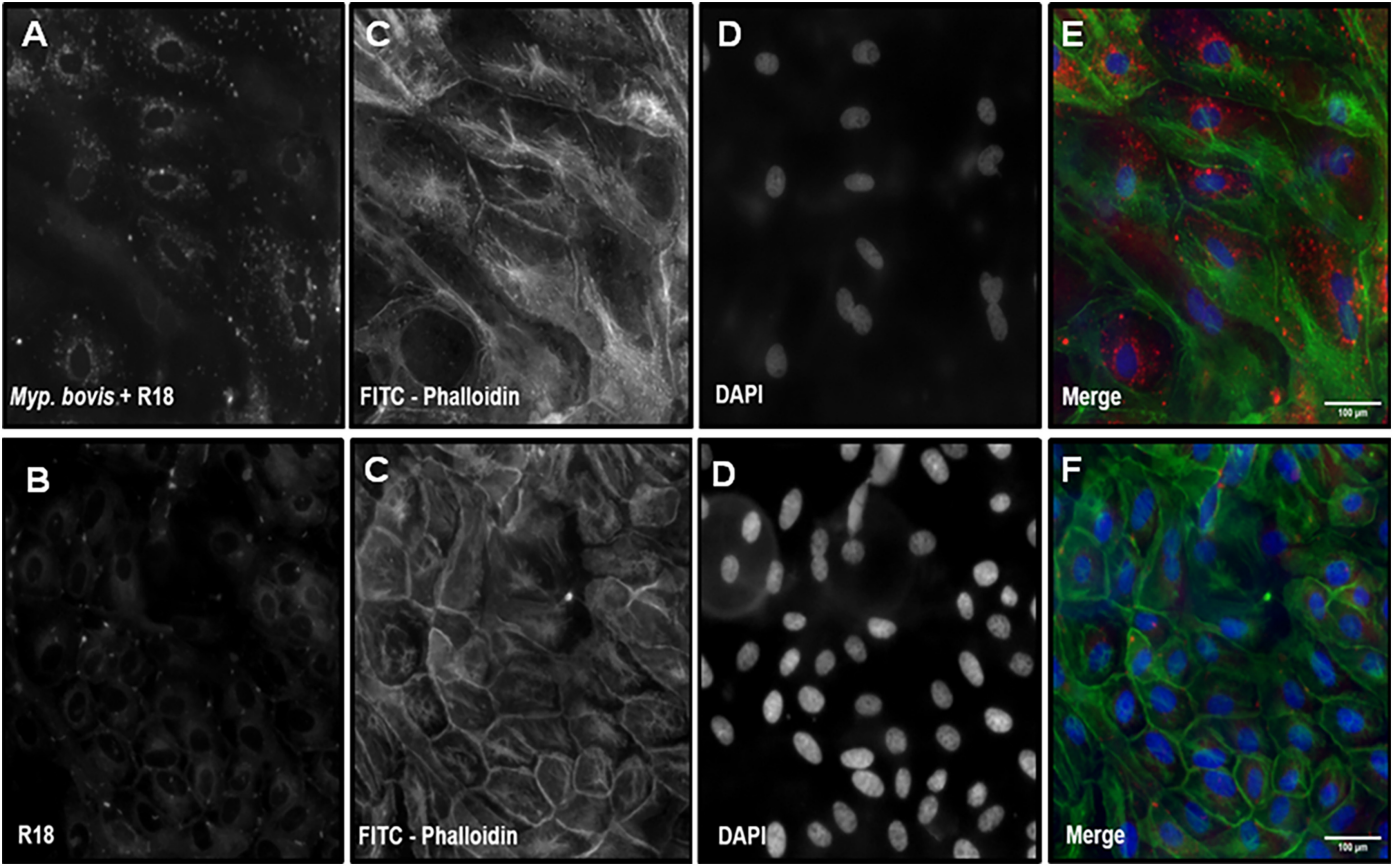
Epi–fluorescent imaging of a co–culture revealed relative positions of *Mycoplasma bovis* (M. bovis) isolate W18_04866 and a bovine endometrial epithelial cell line (bEEL cells). **(A,B)** Represent Octadecyl Rhodamine **(B)** Chloride (R18; 5 μg/mL) staining of *M. bovis* **(A)** including *M. bovis* (pre-stained prior to infection) and **(B)** including Friis Broth + R18 only infection control. Fixed infected and control bEEL cells were post-stained with a green Fluorescein isothiocyanate (FITC)–Phalloidin stain **(C)** (1 ug/mL), and a blue 4′,6–diamidino–2–phenylindole (DAPI) stain **(D)** (2 μg/mL). Images **(A,C,D)** are merged in **(E)**; images **(B–D)** are merged in **(F)**. A 100 μm scale was included.

Epi-fluorescent microscopy of control and co-culture samples confirmed infection of bEEL cells in the presence of R18 dyed *M. bovis* isolate W18_04866 (#1) (Figure 3A). FB was not stained by R18 (Figure 3B). Infected and uninfected bEEL cells demonstrated typical staining patterns for actin (FITC–Phalloidin) (Figure 3C) and nuclei (DAPI) (Figure 3D). Only bEEL cells infected with R18 pre–stained *M. bovis* displayed distinctive areas of punctate–staining (Figure 3E). Proximity of punctate staining to bEEL cell nuclei when imaged through the z-axis likely indicated intracellular infection of *M. bovis*.

### 3.4 Characterisation of isolated sEVs

**FIGURE 4:**
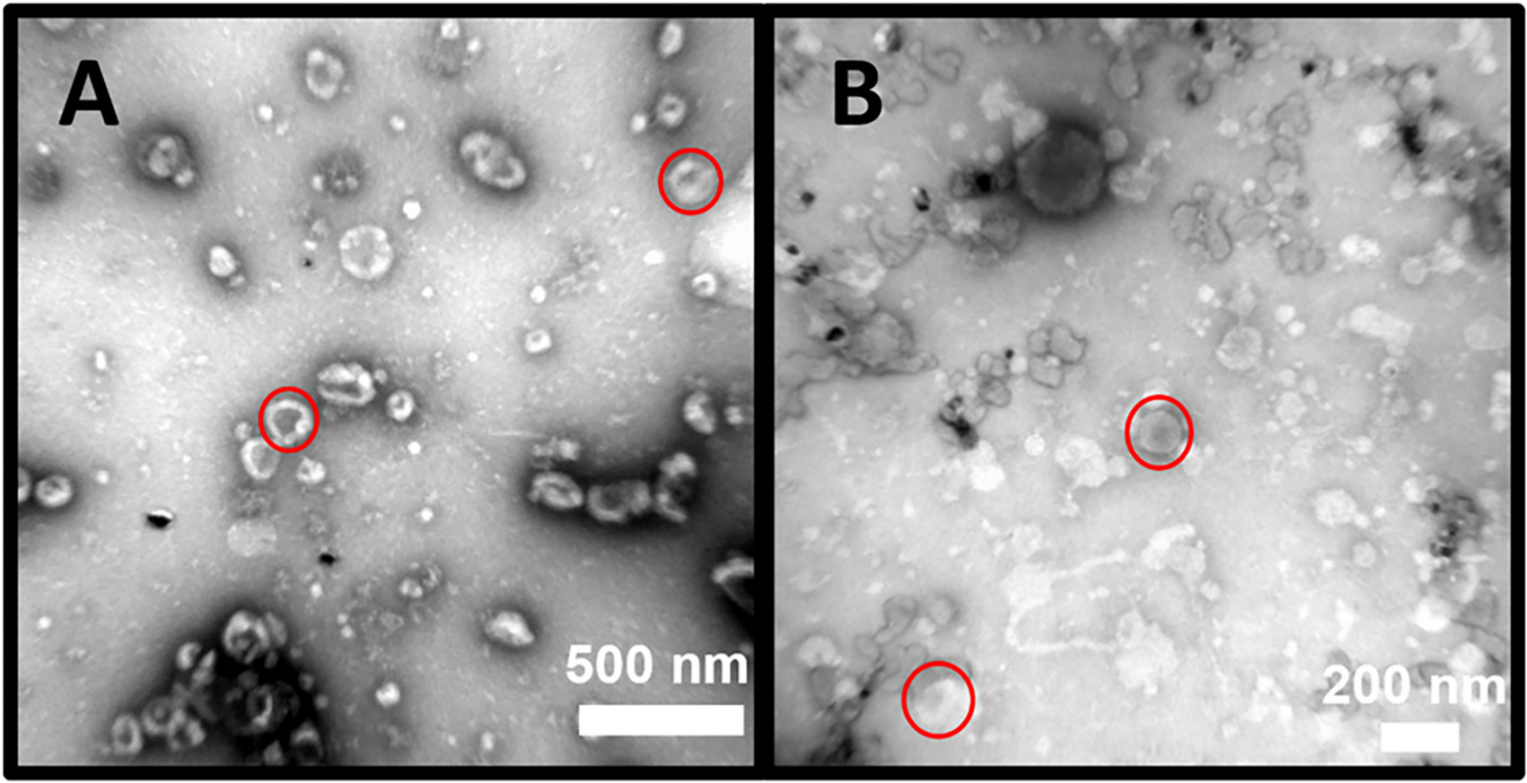
Transmission Electron Microscopy (TEM) revealed a presence of small extracellular vesicles (sEVs) in cell culture of a bioreactor flask. Samples included: clarified medium of a control bovine endometrial epithelial cell line (bEEL cells) bioreactor flask **(A)** and a co–culture bioreactor flask **(B)** containing both bEEL cells and *Mycoplasma bovis* (*M. bovis*) isolate W18_04866 (#1). All images were captured at a magnification of 43,000x. A 500 nm and a 200 nm scale bar were included. Red circles demonstrate cup–shaped vesicles (sEVs).

Consistent with Théry et al. (2018) guidelines, isolated nanoparticles were characterised as sEVs using TEM to determine their shape and size. When negatively stained, sEVs isolated using SEC columns demonstrated a characteristic cup-shape (Figure 4). Evidence of cup–shaped vesicles from control (Figure 4A) and co-culture (Figure 4B) bioreactor harvests confirmed successful isolation of sEVs. The average size range of sEVs was 50–80 nm (Supplementary Figure 1). The size range of co-culture sEVs was comparable to control sEVs when visualised using TEM.

Using NTA and TRPS, sEVs of the control samples had an average size of 177 nm (SD = 29.2 nm, SE = 16.5 nm). Additionally, the average final concentration of the control samples was 3.02 ×x 10^11^ particles/mL (SD = 1.3 × 10^11^ particles/mL, SE = 4.2 × 10^1^ particles/mL).

To provide a reference of *M. bovis* sEV proteins for LC–TIMS-MS/MS, sEVs were also isolated from technical controls of *M. bovis* growth conditions (1.1.3): *M. bovis* cultured in FB and *M. bovis* cultured in aRPMI (supplemented with 4.5% of exosome–depleted FBS, 2x GlutaMAX supplement and 1x Penicillin/Streptomycin).

### 3.5 Using SEC columns, sEVs were isolated to fractions 5–7

**FIGURE 5:**
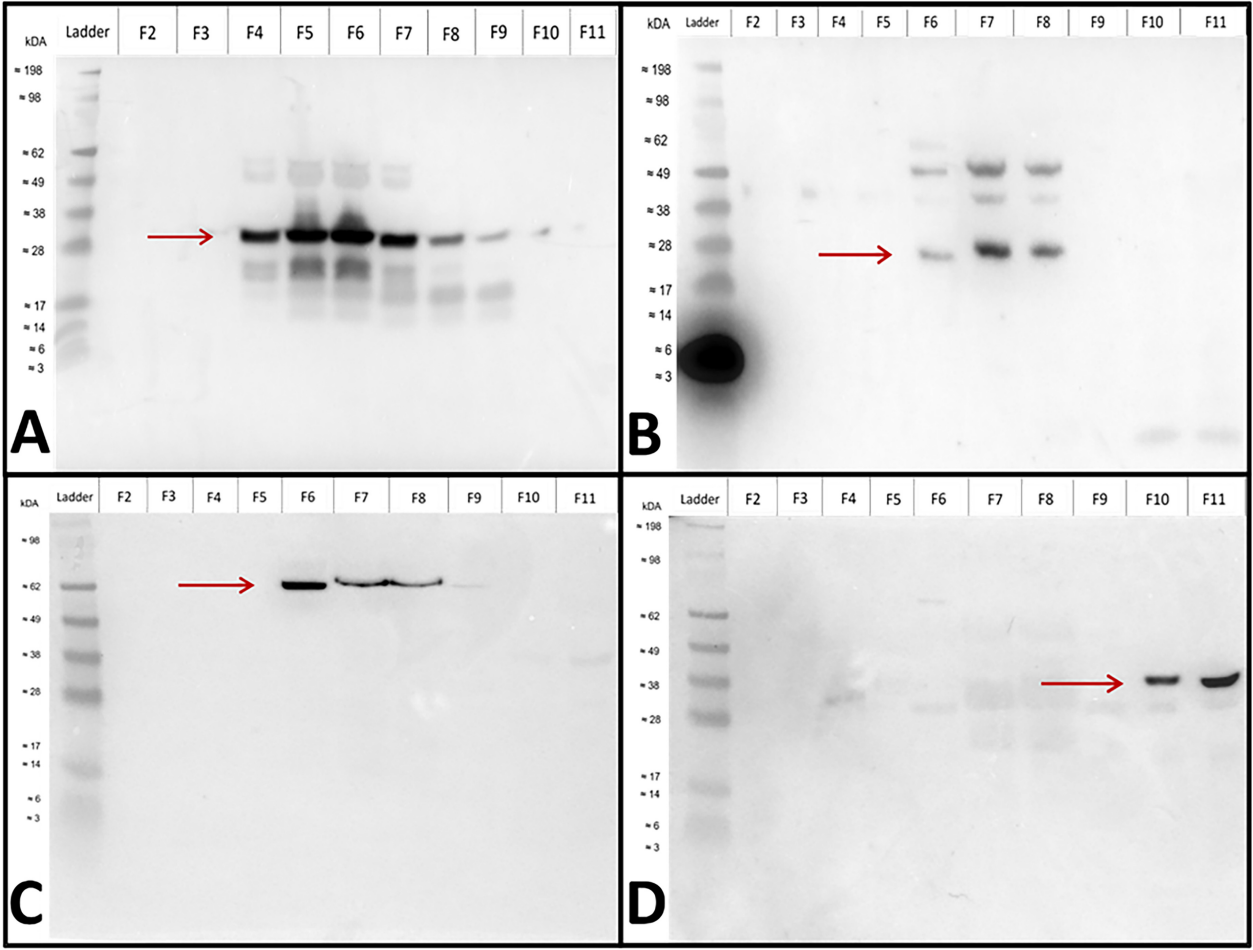
Western blotting revealed proteins associated with small extracellular vesicles (sEVs) using specific/exclusion antibodies. Anti–protein rabbit primary antibodies against Syntenin–1 (expected kDa of ∼32) **(A)** CD63 (expected kDa of ∼26) **(B)**, Heat Shock Protein 70 (expected kDa of 66–78) **(C)**, and Calnexin (expected kDa of ∼98) **(D)**, were diluted 1/1,000 in blocking solution. A goat anti–rabbit horse radish peroxidase secondary antibody, diluted 1/10,000 in blocking solution was used. A SeeBlue Plus2 Pre–stained Protein Standard (Lane 1) was used for size comparison of fractions 2–11 (F2–11).

Western Blot was utilised to verify the presence and positioning of sEVs. In fractions 2–11 (Figure 5), known sEV protein markers were detected. Syntenin–1 was detected in fractions 4–10 at the expected molecular mass of ∼32 kDa (Figure 5A). Proteins bands in fractions 5 and 6 were the strongest intensity, with weaker bands evident in fractions 8, 9, and 10. CD63 was detected in fractions 6–8 at the expected molecular weight of ∼26 kDa (Figure 5B). Protein bands in fraction 7 demonstrated the strongest intensity. Likely, additional bands present at ∼50 kDa were associated with dimerisation of the tetraspanin protein (Zimmerman et al., 2016). Heat–Shock Protein 70 (HSP70) was detected in fractions 6–8 at the expected molecular weight between 66 and 78 kDa (Figure 5C), with the strongest intensity band demonstrated in fraction 6. To prevent potential protein contamination, fraction 8 was not included when fractions were pooled. Calnexin was detected in fractions 10 and 11 at ∼40 kDa, which was smaller than the expected molecular weight of ∼97 kDa (Figure 5D). Protein markers, such as CD63, Syntenin-1, Heat-Shock-70 and Calnexin, were consistent with established inclusion/exclusion sEV markers (Kowal et al., 2016).

### 3.6 Protein profiles of sEV cargo

**FIGURE 6:**
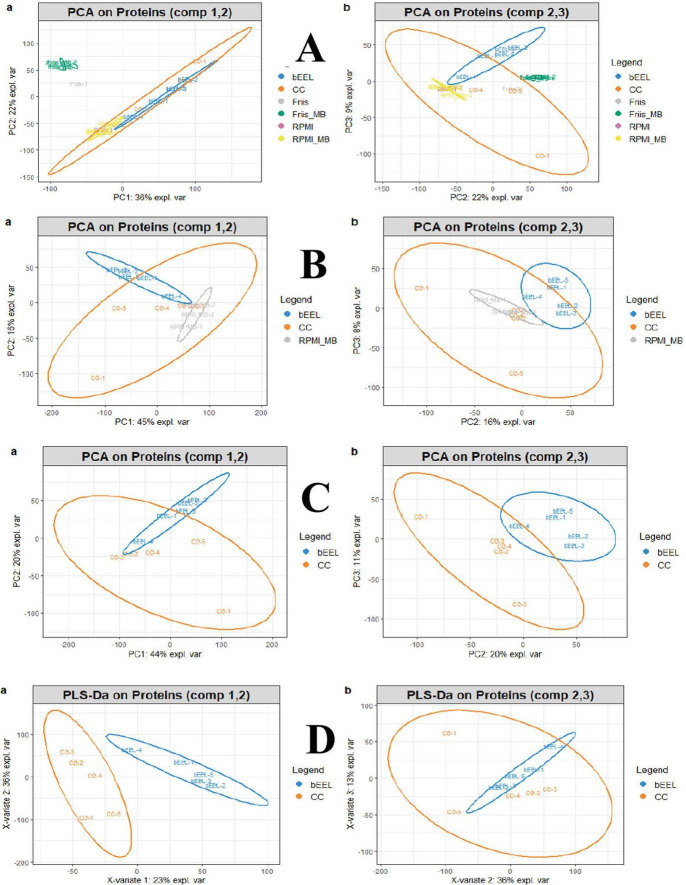
**(A)** Principal component analysis (PCA) of protein abundance from sEVs: blue, bEEL control; orange, co-culture (CC) bEEL + *Mycoplasma bovis* (W18-04866); grey, Friis broth medium-only (Friis); green, *M. bovis* cultured in Friis (Friis_MB); pink, Advanced RPMI medium-only (aRPMI/RPMI); yellow, *M. bovis* cultured in aRPMI (RPMI_MB). **(B,C)** (subset of A; colours as in A excepting RPMI_MB (now grey). **(D)** Partial least-squares discriminant analysis (PLS-DA) of sEV datasets comparing bEEL (blue) and CC (orange). Abbreviations: sEVs (small extracellular vesicles); bEEL (bovine endometrial epithelial cell line).

Overall, 9,807 proteins could be confidently identified across all nineteen samples (5x bEEL control, 5x co-culture, 1x Friis medium only, 4x *M. bovis* grown in Friis, 1x aRPMI medium only, 3x *M. bovis* grown in aRPMI) (Figure 6A). Multivariate analysis, using unsupervised clustering of protein profiles, demonstrated that sEV proteins identified in *M. bovis* grown in FB (Friis_MB) and FB alone (Friis) were clearly separated from other sample types (Figure 6A). As expected, in co-culture harvests there was greater biological variation between samples when compared to control bEEL samples or to *M. bovis* grown in aRPMI (RPMI_MB) samples (Figure 6B). Additionally, sEV proteins identified in control bEEL samples or RPMI_MB samples could be separated using all three components whilst co-culture sEV samples overlapped both sample types (Figure 6B). PCA revealed that the sEV protein profiles from bEEL control and co-culture samples could not be completely distinguished (Figure 6C). However, applying supervised clustering through partial least square discriminant analysis (PLS-DA) enabled discrimination between bEEL control and co-culture samples based on their overall sEV protein profiles (Figure 6D).

**FIGURE 7:**
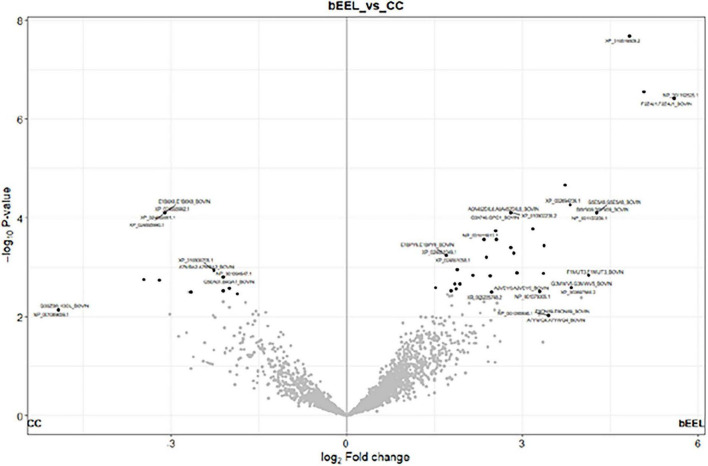
Volcano plot of differential abundance of Bos taurus (Host) proteins (*p* < 0.05 (- log10 adjusted *p*-value > 2) and a log2 (fold change) > 1) from small extracellular vesicles (sEVs). Legend expanded: right hand side (bEEL) denotes proteins extracted from small extracellular vesicles (sEVs) of a bovine endometrial cell line (bEEL) control, left hand side (CC) denotes proteins extracted from sEVs of a co-culture of bEEL cells and *Mycoplasma bovis* (M. bovis) isolate W18-04866. The grey points represent proteins without significant changes, and the black dots represent the upregulated or downregulated proteins compared to the control group.

**FIGURE 8:**
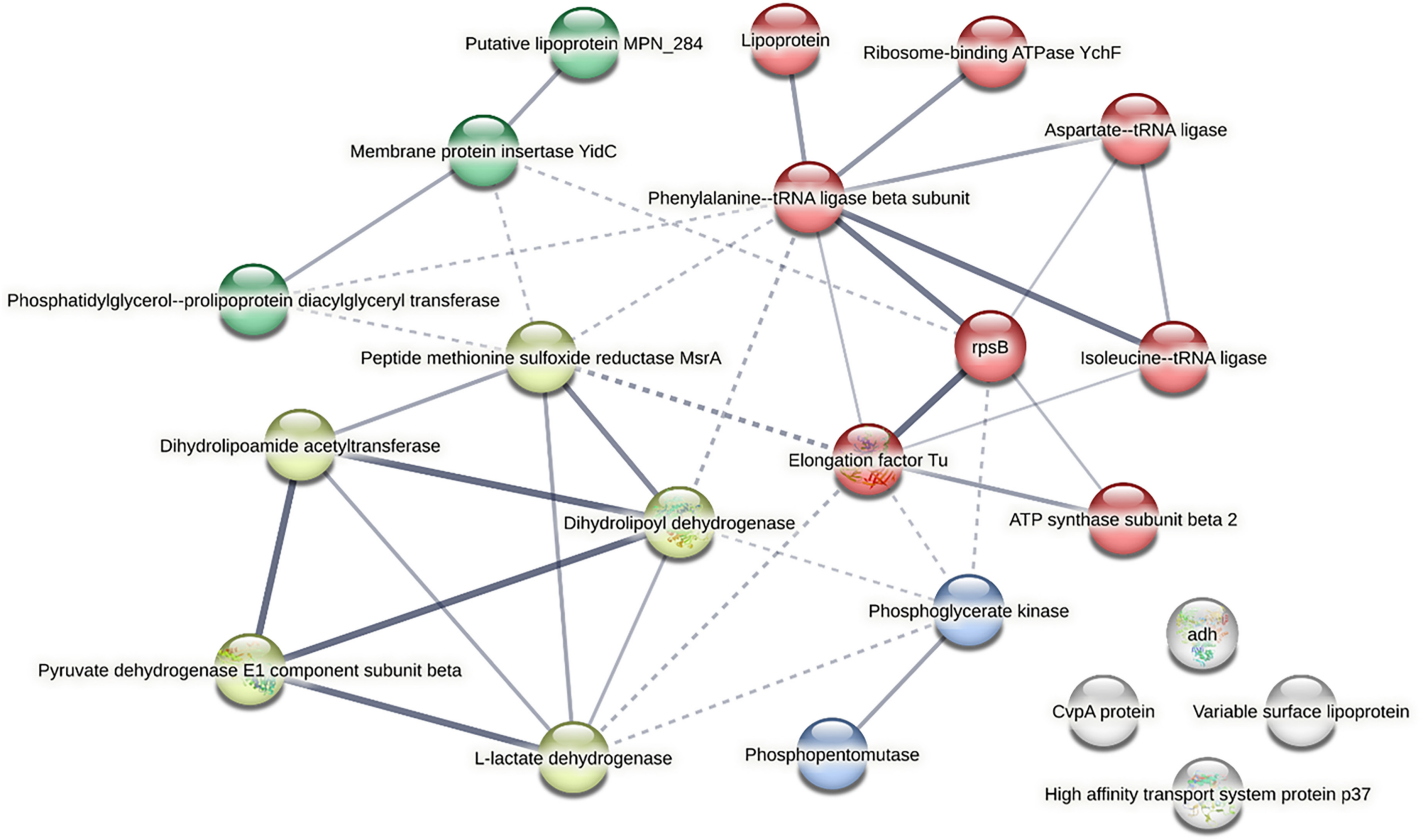
Twenty-seven significant *Mycoplasma bovis* proteins (*p* < 0.05) were input into STRING (https://string-db.org/), with databases available for twenty-two proteins, for formation of a STRING protein–protein interaction (PPI) network (PPI enrichment *p*-value: 0.109). Nodes represent proteins; edges represent high-confidence functional associations in STRING (not necessarily direct binding). The network contains 22 nodes and 36 edges, with an average node degree of 3.27 (average interactions per protein) and an average local clustering coefficient of 0.522. K-means clustering found a defined number of clusters (4) based on their centroids. Edge colour saturation scales with the STRING confidence score. Edges between clusters are represented as a dotted line. Cluster 1 (red) (gene count = eight) is described as protein biosynthesis, cluster 2 (yellow) (gene count = 5) is described as the citric acid cycle, cluster 3 (green) (gene count = 3) is described as Lipoprotein, and cluster 4 (light blue) (gene count = 2) contained Phosphoglycerate kinase and Phosphopentomutase. Generated using STRING (Global Biodata Coalition and ELIXIR). Databases not available for Multifunctional lipase MilA, DUF3137 domain-containing protein, Putative NADP-dependent isopropanol dehydrogenase, 2-oxoisovalerate dehydrogenase subunit alpha and Probable cytosol aminopeptidase.

Next, univariate analysis was applied to identify which sEV proteins were differentially abundant between control (bEEL-only) and co-culture vesicles. Results were visualised using a volcano plot (Figure 7). From the overall dataset of 9,807 proteins, 193 proteins were assessed as being differentially abundant (*p* < 0.05 (*q*-value = -log10 adjusted *p* > 2 and a log2 (fold change) > 1) between the control bEEL and co-culture groups (Supplementary Table 1). Of *Bos taurus* host proteins, Histone 2A and its isoforms had the greatest differential abundance (*q*-value = 1.60 × 10^–13^ with a fold change of 5.59–5.07) in control sEVs relative to co-culture sEVs. Histone 4 was identified as greatly differentially abundant (*q*-value = 1.60 × 10^–13^ with a fold change of 4.83). Fibronectin was identified as greatly differentially abundant (*q*-value = 8.28 × 10^–5^ with a fold change of 4.26–4.26). In contrast, in co-culture sEVs relative to control sEVs, the greatest differentially abundant (*q*-value = 0.0262 with a fold change of −4.92) *Bos taurus* sEV protein was Histone H3.3. Twenty-seven *M. bovis*-associated sEV proteins (Supplementary Table 2) were identified as having greater abundance (*q*-value < 0.05) in co-culture sEVs relative to control sEVs. Databases of twenty-two proteins were available for protein-protein functional associations, which are represented in Figure 8.

## 4 Discussion

Our study has confirmed that a New Zealand isolate of *M. bovis* can be successfully co-cultured with bEEL cells, consistent with other *M. bovis* co-cultures using different cell lines (Bürgi et al., 2018; Josi et al., 2018). There is a tendency for *M. bovis* infection studies to focus upon respiratory or mammary cells as bronchopneumonia or mastitis are major clinical signs for *M. bovis* (Calcutt et al., 2018). However, increasing understanding of endometrial inflammation in dairy cows is essential to prevent increased culling of animals made infertile by *M. bovis* infection (Guo et al., 2014). Utilising bEEL cells provided novel evidence that bovine endometrial epithelial cells are infected by *M. bovis*, with their expression altered by infection. Stability of our co-culture in a static bioreactor flask was clear, with only the initial inoculation of *M. bovis* required to enable at least 3 months of successful co-culturing with bEEL cells. Utilising quantitative PCR for increased detection sensitivity (Andrés-Lasheras et al., 2020) would have been beneficial for specifically assessing *M. bovis* load in control and co-culture bioreactor flasks; however, using a commercial PCR kit had the added benefit of ensuring there was an absence of nineteen common contaminating *Mycoplasma* spp. in the control bioreactor flask, as contaminant *Mycoplasma* spp. would have greatly affected the comparison of sEV proteomes. Future work will incorporate qPCR to quantify bacterial burden and assess whether bacterial load correlates with sEV yield and proteomic changes over time.

*Mycoplasma bovis* virulence mechanisms are still debated, with discussion of *M. bovis* achieving both adherence and invasion in its lifecycle. Bürki et al. (2015b) and van der Merwe et al. (2010) suggest that adherence of *M. bovis* to host cells provides the initial means for intracellular invasion. Many variable surface adhesin proteins exist within the outer membrane of *M. bovis* cells, supporting that adherence to host cells is an integral part of *M. bovis* parasitism (Chen et al., 2018). Z-stacked epi-fluorescent microscopy revealed a tendency of punctate staining surrounding the nucleus of bEEL cells. As the punctate staining was not spread throughout the cytoplasm, it suggested that intracellular invasion of bEEL cells by R18-stained *M. bovis* had occurred. Additionally, a eukaryotic host cell (bEEL cells) was necessary to protect *M. bovis* from consequences caused by increasing gentamicin concentrations, which was consistent with other gentamicin protection assays (Sharma and Puhar, 2019). Whilst Yimthin et al. (2025) demonstrated protection of *Mycoplasma mycoides* subsp. *capri* by bovine macrophages at a gentamicin concentration of 400 μg/mL, VanCleave et al. (2017) demonstrated that peritoneal macrophages provided minimal protection for *Yersinia pestis* as growth was prevented at gentamicin concentrations as low as 4 μg/mL. There is an apparent connection between the eukaryotic cell type and the level of protection it provides intracellular pathogens against an aminoglycoside antibiotic, likely explaining the inability of bEEL cells to protect *M. bovis* from 200 μg/mL of gentamicin. Intriguingly, Nishi et al. (2021) provided evidence demonstrating intracellular invasion of *M. bovis*, which was mediated by the clathrin-dependent endocytosis pathway; a pathway directly involved in early endosome formation and sEV generation (Yoshida et al., 2018). These results provide further evidence toward understanding the extracellular adherence/intracellular infection lifecycle of *M. bovis*.

Our study has described a high-yield/purity method of producing sEVs from a co-culture infection model by combining bioreactor flasks (for increased sEV output) with SEC columns (for improved sEV isolation) (Lobb et al., 2015; Contreras et al., 2023). Utilising a 3D co-culture approach provided advantages in cell morphology that a 2D co-culture system would not (Kapałczyńska et al., 2018), whilst still producing sEVs that were cup-shaped, sized between 50 and 180 nm and contained known sEV marker proteins (Théry et al., 2018). Confirming that the co-culture model reliably generated viable sEVs enabled investigation of changes in the sEV proteome of *Bos taurus* host cells in response to infection by *M. bovis*. The ten most abundant *Bos taurus* proteins in the sEV proteome of uninfected bEEL cells, relative to infected cells, were Histone 2A and its isoforms (Supplementary Table 1). Histone 2A is a central component in maintaining nucleosome integrity, has a vital role in regulating transcription and aids in enabling DNA repair (Dunjić et al., 2023). Cells actively package histones into sEVs to maintain cellular homeostasis and enable intercellular communication (Muthukrishnan, 2018). Presence of extracellular histones through sEV exocytosis triggers neutrophils into forming neutrophil extracellular traps (NETs) when stimulated. NETs are formed through synergism of histones and antimicrobial peptides (AMPs) (Brinkmann et al., 2004); Histone 2A is a key component of NETs. Histones are unable to destroy microbes without aid of AMPs *in vivo* conditions (Doolin et al., 2020), with expressed Histone 2B requiring a binding partner to prevent cell toxicity *in vitro* (Kawasaki et al., 2008). Histone 2A acts to stabilise pores formed in the cell membrane by AMPs and, once able, will directly regulate pathogen transcription thus killing the cell (Doolin et al., 2020). A recent study demonstrated that bacteria such as *M. bovis* that do not have a cell wall, and therefore no lipopolysaccharide, are more susceptible to NETs than those with a cell wall (Duong et al., 2025).

Histone 4 was also of greater abundance in the sEV proteome of control cells relative to infected cells (Supplementary Table 1). As a pathogen defence mechanism, invertebrates innately release extracellular histones from haemocytes, which function similar to those incorporated in NETs of vertebrates (Poirier et al., 2014). Intriguingly, application of both oyster and shrimp-derived Histone 4 demonstrated reduced viability of gram negative bacteria (gram positive bacteria were unaffected) meaning H4 has an essential role in innate immunity in some invertebrates as it reduced bacterial pathogenesis (Dorrington et al., 2011; Yang et al., 2022). To extend its own pathogenesis, *M. bovis* must regulate histone availability to prevent cell stress. Singh et al. (2025) demonstrated stressed cells release more sEVs with capacity to transport histones than control cells. As infection causes cell stress, some cells utilise sEV secretion pathways to present antigens to immunomodulatory cells (Menay et al., 2024). It is well known that intracellular pathogens, such as *M. bovis*, manipulate their host cell by altering transcription pathways (Bush and Rosenbusch, 2004). By disrupting packaging of Histone 2A and Histone 4 into sEVs, *M bovis* reduces presence of a cellular stress indicator and reduces capacity of immunomodulatory cells to form functional clearance mechanisms such as NETs.

Host histone biology is an important component of *M. bovis* pathogenesis, with histone proteins disrupted in different ways. Although Histone 2A was the most differentially expressed host protein in control sEVs, in co-culture conditions, the most abundant protein in the sEV proteome of infected bEEL cells, relative to control uninfected cells, was Histone H3.3C-like protein (Supplementary Table 1). Histone H3.3 is one part of the nucleosome complex and is distributed in actively transcribed chromatin regions (Mito et al., 2007). Post translational modifications affect the N-terminal and C-terminal tails of Histone 3.3, with active gene transcription altered according to cell requirements (Chen et al., 2013). Intracellular pathogens can hijack these mechanisms, adjusting transcription rates according to their own needs for host cell viability. For example, intracellular infection by *Listeria monocytogenes* (*L. monocytogenes*) induces Histone H3 deacetylation. Paired with activated Sirtuin 2 activity, H3 deacetylation modulates DNA damage caused by *L. monocytogenes*, enabling continued host cell survival and *L. monocytogenes* pathogenesis (Eldridge and Hamon, 2021). Removal of Histone 3.3 in sEVs produced by *M. bovis* infected cells may maintain genome stability. By actively removing Histone 3.3, infected host cells prevent an open chromatin structure; therefore, reducing active gene transcription (Szenker et al., 2011) and potential DNA damage induced by *M. bovis*. Alternatively, Histone 3.3 released from infected cells *in vivo* may lead to formation of macrophage extracellular traps (METs), which have been demonstrated as effective microbial destroyers (Baz et al., 2024). Overall, histones play a key role in innate immunity, and it was clear that *M. bovis* infection was responsible for changes in histone shuttling by sEVs. These findings allude that the histone load of sEVs from suspect infected animals could be compared to known healthy animals to indicate asymptomatic carriers, which is important for diagnostic testing of herds to prevent continual spread of disease.

An improvement to current diagnostic testing may be the inclusion of some of the twenty-seven *M. bovis*-associated sEV proteins detected in co-culture sEVs relative to control sEVs (Supplementary Table 2). Two proteins of note were Mycoplasma immunogenic lipase A (MilA) and p80 family lipoprotein. MilA is predicted as a lipase responsible for hydrolysis of lipids, aiding nutritional requirements of *Mycoplasma* spp. (Adamu et al., 2020). Lipoproteins in *Mycoplasma* spp. are multifunctional and are involved in secretion, adhesion and modulation of host-immune responses (Christodoulides et al., 2018). Lipoproteins have been identified as potential controls for *M. bovis* infection, as they function within host cells as nucleomodulins. Recent functional studies have identified novel lipoproteins capable of inducing an inflammatory cytokine response in host cells (Lu et al., 2021; Yuan et al., 2025) and inducing apoptosis of a macrophage cell line (Zhao et al., 2021; Raheem et al., 2024). Whilst previous research has explored the potential of variable surface lipoproteins (Lysnyansky et al., 1999), recent secretome studies revealed novel lipoproteins secreted by intracellular *M. bovis* that influence pathogenesis (Zhang et al., 2021; Zhao et al., 2022). As both surface and secreted lipoproteins are encapsulated within host sEVs (Zou et al., 2022), increasing the availability of extracellular virulence factors to the host innate immune system will trigger cytokine activation pathways that lead to beneficial monocyte activity (Lu et al., 2021). Recombinant versions of MilA and MbovP579 (homolog of membrane lipoprotein p81 or p80) were successfully utilised in indirect ELISAs to detect *M. bovis* in sera of infected bovine animals (Wawegama et al., 2014; Khan et al., 2016). More importantly, these recombinant proteins were *M. bovis* specific and were able to distinguish *M. bovis* from other closely related *Mycoplasma* spp. such as *Mycoplasma agalactiae*. Sensitivity and specificity of current *M. bovis* diagnostic testing is affected by the antigenic shift of the most immunogenic proteins, variable surface proteins (Bürki et al., 2015a). Therefore, detection of immunoreactive proteins that are limited in their phase and antigenic variation are very useful for their diagnostic potential. Researchers should explore these proteins further as diagnostic markers for *M. bovis* as their presence in sEVs of infected cells supports previous experiments exploring *M. bovis* pathogenesis.

Two major groupings were revealed when their protein-protein functional associations were k-means clustered. The greatest predicted confidence of contributing to a shared function was between dihydrolipoamide acetyltransferase (E2), dihydrolipoyl dehydrogenase, pyruvate dehydrogenase, pyruvate dehydrogenase E1 component subunit beta (E1) and L-lactate dehydrogenase. Each of these proteins (excluding L-lactate dehydrogenase) forms a functional unit of the pyruvate dehydrogenase complex (PDC), an essential catalytic complex that links glycolysis to the tricarboxylic acid (TCA) cycle (Patel et al., 2014). Whilst not specifically associated with the PDC, L-lactate dehydrogenase catalyses the reversible conversion of pyruvate to lactate to enable energy production in anaerobic environments (Brooks et al., 1999). These proteins are universal as all organisms that utilise mitochondria for energy conversion contain this complex. However, *M. bovis* does not contain certain components essential in the TCA cycle, instead reliant on its host and surface-exposed glycolytic enzymes for energy conversion (Masukagami et al., 2017). Instead, some glycolytic enzymes play diverse roles in *Mycoplasma* spp. with a few identified as adhesion proteins that can interact with host cell components. Ma et al. (2024) demonstrated that E2 is essential in mediating adhesion and invasion of an avian pathogen, *Mycoplasma synoviae* (*M. synoviae*), to host cells. In support, Qi et al. (2022) revealed that dihydrolipoamide dehydrogenase (DLD) (another crucial component of the PDC) of *M. synoviae* played a role in adhesion and pathogenesis of host cells. Additionally, fibronectin (a large glycoprotein essential for modulating cell adhesion, differentiation and growth in a great variety of tissue types (Schwarzbauer and DeSimone, 2011) was bound by *M. synoviae* DLD, which supports evidence of reduced fibronectin abundance in *M. bovis* co-culture sEVs as it is likely host fibronectin was bound by infectious *M. bovis* as compared to uninfected bEEL cells. Whilst understanding of the use of E1 and L-lactate dehydrogenase as *M. bovis* virulence factors remain limited, with research focused on exploring their localisation and host interactions (Gründel et al., 2016); intriguingly, Sun et al. (2014) demonstrated the potential of E1 as a ELISA marker for *M. bovis* infection as there was no reaction with other pathogens tested excepting *Mycoplasma agalactiae*.

Host and pathogen-derived proteins identified in sEVs represent promising candidates for inclusion in multiplex diagnostic panels. Integrating these targets into existing testing workflows may enhance sensitivity and specificity, particularly for detecting subclinical infections. Future research should validate these sEV-associated proteins using larger sample sets, clinical specimens, and multi-analyte assays to deliver practical diagnostic solutions for surveillance and management. Together, sEV proteins provide valuable insight into *M. bovis* virulence mechanisms and demonstrate that changes in the sEV proteome of host cells result from *M. bovis* infection.

## 5 Conclusion

This study explored a novel technique of combining a bovine *in vitro* infection model with a 3D static bioreactor flask to compare the protein cargo of sEVs generated from control and co-culture conditions. Whilst a novel biomarker of *M. bovis* infection was not determined, the host cell sEV proteome profile indicated *M. bovis* manipulated histone presentation and affected nucleosome integrity. The presence of different histones, depending on the culture condition, in the sEV proteome was a direct influence of *M. bovis* infection. By reducing presence of cell stress indicators, *M. bovis* reduces the host’s ability to establish proper microbial defence mechanisms, therefore reducing pathogen detection and removal by host cells. Notably, a reduction in host adhesion proteins, such as fibronectin, suggested *M. bovis* prevented host protein availability through intracellular invasion. Many *M. bovis*–associated sEV proteins aligned with bacterial metabolism; however, enrichment of glycolytic enzymes with established multifunctionality in *Mycoplasma* spp. suggests an involvement in pathogenesis and warrants further investigation.

Importantly, previously reported immunoreactive *M. bovis* proteins were present in sEVs of infected cells, which supports the possibility of an improved diagnostic test for early detection of *M. bovis* using sEVs. Further, these results provide evidence that infection of host cells is a multifaceted process, with cellular adhesion and intracellular invasion integral to the persistence of *M. bovis*. Whilst the proteins identified in co-culture sEVs did not elucidate novel biomarker candidates for a diagnostic test, they offer valuable insights into sEV cargo during bacterial infection and provide further evidence to understand *M. bovis* pathogenesis.

## Data Availability

The datasets presented in this study can be found in online repositories. The names of the repository/repositories and accession number(s) can be found in the article/Supplementary material.
