# Supplementary material for: Mycoplasma bovis infection alters small extracellular vesicle cargo derived from bovine endometrial epithelial cells cultured in static bioreactors
Source: Front Microbiol. 2026 May 7;17:1770401. doi: 10.3389/fmicb.2026.1770401 (PMC13190619; doi:10.3389/fmicb.2026.1770401)
Supplement: Supplementary file 1 [file Table_1.docx]

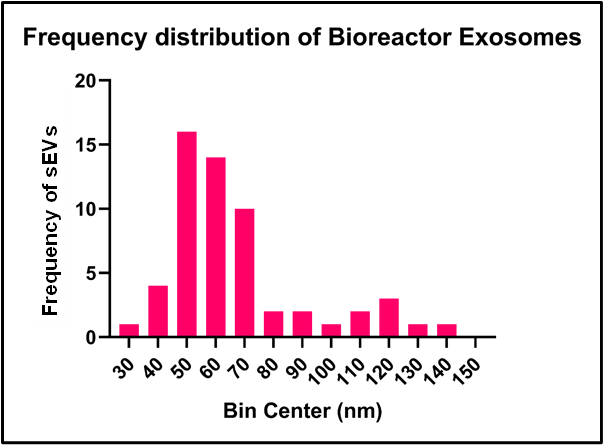


Supplementary Figure 1: Size distribution of small extracellular vesicles (sEVs) isolated from the bovine endometrial epithelial cell line (bEEL cells) control bioreactor (uninfected cells). sEV diameters were measured manually from five Transmission Electron Microscopy (TEM) images at a magnification of 43,000x and 135,000x, with a minimum of 50 vesicles measured per image. The average sEV size range was 50–80 nm, consistent with established sEV classifications.

Supplementary Table 2: Significant Mycoplasma bovisproteins (p-value <0.05 (-log10 adjusted p-value >2) and a log2 (fold change) >1) from small extracellular vesicles (sEVs). Legend expanded: (bEEL) denotes proteins extracted from small extracellular vesicles (sEVs) of a bovine endometrial cell line (bEEL) control, CC denotes proteins extracted from sEVs of a co-culture of bEEL cells and Mycoplasma bovis(M. bovis) isolate W18-04866.

| **ID** | **Protein name** | **Gene** | **Organism** | **bEEL_vs_CC_p.val** | **bEEL_vs_CC_p.adj** | **bEEL_vs_CC_ratio** | **bEEL centered** | **CC centered** |
| --- | --- | --- | --- | --- | --- | --- | --- | --- |
| A0A2N8U1F6.A0A2N8U1F6_MYCBV | Phenylalanine--tRNA ligase beta subunit | pheT | *Mycoplasma bovis* | 0.0138033 | 0.0146 | 4.43 | 2.21 | -2.21 |
| A0A2N8U288.A0A2N8U288_MYCBV | Ribosome-binding ATPase YchF | ychF | *Mycoplasma bovis* | 0.0005007 | 0.000155 | 3.91 | 1.3 | -2.61 |
| A0A2N8U351.A0A2N8U351_MYCBV | Phosphoglycerate kinase | pgk | *Mycoplasma bovis* | 0.0002149 | 0.000665 | 3.62 | 1.81 | -1.81 |
| A0A2N8U234.A0A2N8U234_MYCBV | Aspartate--tRNA ligase | aspS | *Mycoplasma bovis* | 0.0011261 | 0.0119 | 2.44 | 1.22 | -1.22 |
| A0A2N8U253.A0A2N8U253_MYCBV | Multifunctional lipase Mycoplasma immunogenic lipase A (MilA) | milA | *Mycoplasma bovis* | 0.0023836 | 0.0351 | 1.56 | 0.78 | -0.78 |
| A0A2N8U2Q5.A0A2N8U2Q5_MYCBV | DUF3137 domain-containing protein | HYD69_01635 | *Mycoplasma bovis* | 0.0017935 | 0.0243 | -2.4 | -1.2 | 1.2 |
| A0A2N8U1V9.A0A2N8U1V9_MYCBV | CvpA family protein | MBOVJF4278_00174 | *Mycoplasma bovis* | 0.0041139 | 0.0364 | -2.51 | -1.43 | 1.07 |
| A0A1B0Z6M0.A0A1B0Z6M0_MYCBV | Dihydrolipoyl dehydrogenase | pdhD | *Mycoplasma bovis* | 0.0003722 | 0.0019 | -2.78 | -1.39 | 1.39 |
| A0A2N8U2G5.A0A2N8U2G5_MYCBV | Peptide methionine sulfoxide reductase MsrA | msrB | *Mycoplasma bovis* | 0.007751 | 0.0479 | -2.8 | -1.68 | 1.12 |
| A0A2N8U224.A0A2N8U224_MYCBV | Putative NADP-dependent isopropanol dehydrogenase or Zinc-dependent alcohol dehydrogenase family protein | HYD69_02450 | *Mycoplasma bovis* | 0.004784 | 0.0449 | -2.94 | -1.26 | 1.68 |
| A0A2N8U1Y8.A0A2N8U1Y8_MYCBV | Isoleucine--tRNA ligase | ileS | *Mycoplasma bovis* | 0.0004062 | 0.000737 | -2.96 | -1.48 | 1.48 |
| A0A2N8U230.A0A2N8U230_MYCBV | Small ribosomal subunit protein uS2 | rpsB | *Mycoplasma bovis* | 0.0043603 | 0.0394 | -3.06 | -1.31 | 1.75 |
| A0A2N8U1F3.A0A2N8U1F3_MYCBV | Membrane protein insertase YidC | yidC | *Mycoplasma bovis* | 0.0001622 | 0.000166 | -3.16 | -1.41 | 1.76 |
| A0A2N8U2Q7.A0A2N8U2Q7_MYCBV | Phosphopentomutase | deoB | *Mycoplasma bovis* | 0.0012008 | 0.0132 | -3.25 | -1.63 | 1.63 |
| A0A2N8U256.A0A2N8U256_MYCBV | alcohol dehydrogenase | adh | *Mycoplasma bovis* | 0.0002588 | 0.000986 | -3.67 | -1.84 | 1.84 |
| A0A2N8U1G9.A0A2N8U1G9_MYCBV | Phosphatidylglycerol--prolipoprotein diacylglyceryl transferase | lgt | *Mycoplasma bovis* | 0.0028835 | 0.0128 | -3.86 | -3.22 | 0.643 |
| A0A2N8U2T2.A0A2N8U2T2_MYCBV | P80 family lipoprotein or Putative lipoprotein MPN_284 | HYD69_01350 | *Mycoplasma bovis* | 0.000131 | 0.000205 | -4.07 | -2.04 | 2.04 |
| A0A193CK90.A0A193CK90_MYCBV | 2-oxoisovalerate dehydrogenase subunit alpha or Branched-chain alpha-keto acid dehydrogenase E1 component alpha chain | BC94_0086 | *Mycoplasma bovis* | 1.26E-05 | 1.89E-07 | -4.24 | -2.12 | 2.12 |
| A0A2N8U2L3.A0A2N8U2L3_MYCBV | ATP synthase subunit beta 2 | MBOVJF4278_00494 | *Mycoplasma bovis* | 0.0014929 | 0.0101 | -4.28 | -2.68 | 1.61 |
| A0A193CK94.A0A193CK94_MYCBV | Pyruvate dehydrogenase E1 component subunit beta | PDHB | *Mycoplasma bovis* | 9.30E-05 | 9.88E-05 | -4.33 | -2.16 | 2.16 |
| A0A2N8U273.A0A2N8U273_MYCBV | ABC transporter substrate-binding protein or High affinity transport system protein p37 | SBP | *Mycoplasma bovis* | 0.0012316 | 0.00739 | -4.39 | -2.74 | 1.65 |
| A0A2N8U3J8.A0A2N8U3J8_MYCBV | Probable cytosol aminopeptidase or Leucine aminopeptidase | BC94_0699 | *Mycoplasma bovis* | 0.0001095 | 6.30E-05 | -4.61 | -2.56 | 2.05 |
| A0A2N8U2Q4.A0A2N8U2Q4_MYCBV | L-lactate dehydrogenase | HYD69_01415 | *Mycoplasma bovis* | 1.93E-05 | 8.10E-07 | -4.61 | -2.31 | 2.31 |
| A0A2N8U3I7.A0A2N8U3I7_MYCBV | Variable surface lipoprotein | MBOVJF4278_00818 | *Mycoplasma bovis* | 3.04E-06 | 4.38E-10 | -4.7 | -2.35 | 2.35 |
| A0A2N8U1I5.A0A2N8U1I5_MYCBV | Dihydrolipoamide acetyltransferase component of pyruvate dehydrogenase complex | BC94_0088 | *Mycoplasma bovis* | 1.82E-05 | 6.84E-07 | -4.7 | -2.35 | 2.35 |
| A0A2N8U1H9.A0A2N8U1H9_MYCBV | Lipoprotein | HYD69_00530 | *Mycoplasma bovis* | 7.83E-05 | 6.48E-05 | -5.33 | -2.66 | 2.66 |
| A0A2N8U2M1.A0A2N8U2M1_MYCBV | Elongation factor Tu | tuf | *Mycoplasma bovis* | 7.98E-07 | 2.93E-13 | -5.66 | -2.83 | 2.83 |

Supplementary Table 1: Significant proteins (p-value <0.05 (-log10 adjusted p-value >2) and a log2 (fold change) >1) from small extracellular vesicles (sEVs). Legend expanded: (bEEL) denotes proteins extracted from small extracellular vesicles (sEVs) of a bovine endometrial cell line (bEEL) control, CC denotes proteins extracted from sEVs of a co-culture of bEEL cells and Mycoplasma bovis(M. bovis) isolate W18-04866.

| **ID** | **Protein name** | **Gene** | **Organism** | **bEEL_vs_CC_p.val** | **bEEL_vs_CC_p.adj** | **bEEL_vs_CC_ratio** | **bEEL centered** | **CC centered** |
| --- | --- | --- | --- | --- | --- | --- | --- | --- |
| NP_001192525.1 | Histone H2A | H2AC6 | *Bos taurus* (Bovine) | 4.28E-07 | 1.60E-13 | 5.59 | 2.1 | -3.49 |
| F2Z4J1.F2Z4J1_BOVIN | Histone H2A | H2AC6 | *Bos taurus* (Bovine) | 4.28E-07 | 1.60E-13 | 5.59 | 2.1 | -3.49 |
| XP_005209436.1 | Core histone macro-H2A | MACROH2A1 | *Bos taurus* (Bovine) | 3.04E-07 | 1.60E-13 | 5.07 | 2.26 | -2.82 |
| A0A3Q1LZ47.A0A3Q1LZ47_BOVIN | Core histone macro-H2A | MACROH2A2 | *Bos taurus* (Bovine) | 3.04E-07 | 1.60E-13 | 5.07 | 2.26 | -2.82 |
| XP_005209434.1 | core histone macro-H2A.1 isoform X1 | MACROH2A2 | *Bos taurus* (Bovine) | 3.04E-07 | 1.60E-13 | 5.07 | 2.26 | -2.82 |
| XP_005209433.1 | core histone macro-H2A.1 isoform X1 | MACROH2A2 | *Bos taurus* (Bovine) | 3.04E-07 | 1.60E-13 | 5.07 | 2.26 | -2.82 |
| NP_001039805.1 | core histone macro-H2A.1 | MACROH2A2 | *Bos taurus* (Bovine) | 3.04E-07 | 1.60E-13 | 5.07 | 2.26 | -2.82 |
| XP_005209435.1 | core histone macro-H2A.1 isoform X3 | MACROH2A2 | *Bos taurus* (Bovine) | 3.04E-07 | 1.60E-13 | 5.07 | 2.26 | -2.82 |
| XP_024850109.1 | core histone macro-H2A.1 isoform X2 | MACROH2A2 | *Bos taurus* (Bovine) | 3.04E-07 | 1.60E-13 | 5.07 | 2.26 | -2.82 |
| Q2HJ65.Q2HJ65_BOVIN | Core histone macro-H2A | MACROH2A1 | *Bos taurus* (Bovine) | 3.04E-07 | 1.60E-13 | 5.07 | 2.26 | -2.82 |
| XP_010816809.2 | histone H4-like | H4 | *Bos taurus* (Bovine) | 2.33E-08 | 1.60E-13 | 4.83 | 2.41 | -2.41 |
| A0A2N8U1F6.A0A2N8U1F6_MYCBV | Phenylalanine--tRNA ligase beta subunit | pheT | *Mycoplasma bovis* | 0.0138033 | 0.0146 | 4.43 | 2.21 | -2.21 |
| G5E5A8.G5E5A8_BOVIN | Fibronectin | FN1 | *Bos taurus* (Bovine) | 8.28E-05 | 7.40E-05 | 4.26 | 2.13 | -2.13 |
| B8Y9S9.B8Y9S9_BOVIN | Fibronectin | FN1 | *Bos taurus* (Bovine) | 8.28E-05 | 7.40E-05 | 4.26 | 2.13 | -2.13 |
| NP_001157250.1 | Fibronectin | FN1 | *Bos taurus* (Bovine) | 8.28E-05 | 7.40E-05 | 4.26 | 2.13 | -2.13 |
| F1MUT3.F1MUT3_BOVIN | xanthine dehydrogenase/oxidase | xhd | *Bos taurus* (Bovine) | 0.0014847 | 0.00644 | 4.13 | 2.36 | -1.77 |
| A0A2N8U288.A0A2N8U288_MYCBV | Ribosome-binding ATPase YchF | ychF | *Mycoplasma bovis* | 0.0005007 | 0.000155 | 3.91 | 1.3 | -2.61 |
| XP_002697564.2 | H1.4 linker histone, cluster member | H1-4 | *Bos taurus* (Bovine) | 0.0027182 | 0.0114 | 3.84 | 0.639 | -3.2 |
| G3MWV5.G3MWV5_BOVIN | H1.4 linker histone, cluster member | H1-4 | *Bos taurus* (Bovine) | 0.0027182 | 0.0114 | 3.84 | 0.639 | -3.2 |
| XP_002694239.1 | agrin isoform X2 | LOC100642737 | *Bos taurus* (Bovine) | 5.71E-05 | 1.54E-06 | 3.81 | 1.43 | -2.38 |
| NP_001029795.1 | Synaptogyrin 1 | SYNGR1 | *Bos taurus* (Bovine) | 2.38E-05 | 1.45E-09 | 3.73 | 2.13 | -1.6 |
| A0A3Q1NHB1.A0A3Q1NHB1_BOVIN | Synaptogyrin | SYNGR1 | *Bos taurus* (Bovine) | 2.38E-05 | 1.45E-09 | 3.73 | 2.13 | -1.6 |
| A0A3Q1LMT7.A0A3Q1LMT7_BOVIN | Synaptogyrin 1 | SYNGR1 | *Bos taurus* (Bovine) | 2.38E-05 | 1.45E-09 | 3.73 | 2.13 | -1.6 |
| Q3SZ33.Q3SZ33_BOVIN | Synaptogyrin 1 | SYNGR1 | *Bos taurus* (Bovine) | 2.38E-05 | 1.45E-09 | 3.73 | 2.13 | -1.6 |
| XP_010804016.1 | synaptogyrin-1 isoform X2 | SYNGR1 | *Bos taurus* (Bovine) | 2.38E-05 | 1.45E-09 | 3.73 | 2.13 | -1.6 |
| XP_005207391.1 | synaptogyrin-1 isoform X1 | SYNGR1 | *Bos taurus* (Bovine) | 2.38E-05 | 1.45E-09 | 3.73 | 2.13 | -1.6 |
| A0A2N8U351.A0A2N8U351_MYCBV | Phosphoglycerate kinase | pgk | *Mycoplasma bovis* | 0.0002149 | 0.000665 | 3.62 | 1.81 | -1.81 |
| A7YWQ4.A7YWQ4_BOVIN | Beta-2-syntrophin | SNTB2 | *Bos taurus* (Bovine) | 0.0100477 | 0.0224 | 3.44 | 1.15 | -2.3 |
| F6QN89.F6QN89_BOVIN | Beta-2-syntrophin | SNTB2 | *Bos taurus* (Bovine) | 0.0100477 | 0.0224 | 3.44 | 1.15 | -2.3 |
| NP_001098936.1 | Beta-2-syntrophin | SNTB2 | *Bos taurus* (Bovine) | 0.0100477 | 0.0224 | 3.44 | 1.15 | -2.3 |
| XP_015328045.1 | collagen alpha-1(XV) chain isoform X1 | COL15A1 | *Bos taurus* (Bovine) | 0.0003746 | 0.00192 | 3.37 | 1.68 | -1.68 |
| A0A3Q1MUA3.A0A3Q1MUA3_BOVIN | Collagen type XV alpha 1 chain | COL15A1 | *Bos taurus* (Bovine) | 0.0003746 | 0.00192 | 3.37 | 1.68 | -1.68 |
| XP_024851191.1 | Collagen type XV alpha 1 chain | COL15A1 | *Bos taurus* (Bovine) | 0.0003746 | 0.00192 | 3.37 | 1.68 | -1.68 |
| A0A3Q1M4P8.A0A3Q1M4P8_BOVIN | Collagen type XV alpha 1 chain | COL15A1 | *Bos taurus* (Bovine) | 0.0003746 | 0.00192 | 3.37 | 1.68 | -1.68 |
| XP_005210256.1 | Collagen type XV alpha 1 chain | COL15A1 | *Bos taurus* (Bovine) | 0.0003746 | 0.00192 | 3.37 | 1.68 | -1.68 |
| XP_024851190.1 | Collagen type XV alpha 1 chain | COL15A1 | *Bos taurus* (Bovine) | 0.0003746 | 0.00192 | 3.37 | 1.68 | -1.68 |
| XP_024851192.1 | Collagen type XV alpha 1 chain | COL15A1 | *Bos taurus* (Bovine) | 0.0003746 | 0.00192 | 3.37 | 1.68 | -1.68 |
| F1MUC5.F1MUC5_BOVIN | Collagen type XV alpha 1 chain | COL15A1 | *Bos taurus* (Bovine) | 0.0003746 | 0.00192 | 3.37 | 1.68 | -1.68 |
| A0A3Q1LXM2.A0A3Q1LXM2_BOVIN | Collagen type XV alpha 1 chain | COL15A1 | *Bos taurus* (Bovine) | 0.0003746 | 0.00192 | 3.37 | 1.68 | -1.68 |
| NP_001178214.2 | Collagen type XV alpha 1 chain | COL15A1 | *Bos taurus* (Bovine) | 0.0003746 | 0.00192 | 3.37 | 1.68 | -1.68 |
| A0A3Q1M0N0.A0A3Q1M0N0_BOVIN | Periphilin 1 | PPHLN1 | *Bos taurus* (Bovine) | 0.0003746 | 0.00192 | 3.37 | 1.68 | -1.68 |
| P17697.CLUS_BOVIN | Clusterin | CLU | *Bos taurus* (Bovine) | 0.001372 | 0.0163 | 3.36 | 1.68 | -1.68 |
| XP_005209898.1 | Clusterin | CLU | *Bos taurus* (Bovine) | 0.001372 | 0.0163 | 3.36 | 1.68 | -1.68 |
| XP_024851197.1 | Clusterin | CLU | *Bos taurus* (Bovine) | 0.001372 | 0.0163 | 3.36 | 1.68 | -1.68 |
| NP_776327.1 | Clusterin | CLU | *Bos taurus* (Bovine) | 0.001372 | 0.0163 | 3.36 | 1.68 | -1.68 |
| NP_001075005.1 | Collagen triple helix repeat containing 1 | CTHRC1 | *Bos taurus* (Bovine) | 0.0032548 | 0.0164 | 3.29 | 0.549 | -2.74 |
| A2VDY0.A2VDY0_BOVIN | Collagen triple helix repeat containing 1 | CTHRC1 | *Bos taurus* (Bovine) | 0.0032548 | 0.0164 | 3.29 | 0.549 | -2.74 |
| XP_024856053.1 | supervillin isoform X14 | SVIL | *Bos taurus* (Bovine) | 0.0001734 | 0.00019 | 3.18 | 1.41 | -1.77 |
| XP_024856046.1 | supervillin isoform X9 | SVIL | *Bos taurus* (Bovine) | 0.0001734 | 0.00019 | 3.18 | 1.41 | -1.77 |
| XP_024856042.1 | supervillin isoform X10 | SVIL | *Bos taurus* (Bovine) | 0.0001734 | 0.00019 | 3.18 | 1.41 | -1.77 |
| XP_024856057.1 | supervillin isoform X11 | SVIL | *Bos taurus* (Bovine) | 0.0001734 | 0.00019 | 3.18 | 1.41 | -1.77 |
| XP_024856039.1 | supervillin isoform X9 | SVIL | *Bos taurus* (Bovine) | 0.0001734 | 0.00019 | 3.18 | 1.41 | -1.77 |
| XP_024856041.1 | supervillin isoform X9 | SVIL | *Bos taurus* (Bovine) | 0.0001734 | 0.00019 | 3.18 | 1.41 | -1.77 |
| XP_024856052.1 | supervillin isoform X13 | SVIL | *Bos taurus* (Bovine) | 0.0001734 | 0.00019 | 3.18 | 1.41 | -1.77 |
| XP_024856043.1 | supervillin isoform X9 | SVIL | *Bos taurus* (Bovine) | 0.0001734 | 0.00019 | 3.18 | 1.41 | -1.77 |
| A0A3Q1LXG9.A0A3Q1LXG9_BOVIN | von Willebrand factor A domain-containing protein 1 | VWA1 | *Bos taurus* (Bovine) | 0.0013375 | 0.0116 | 2.9 | 1.29 | -1.61 |
| NP_001096700.1 | von Willebrand factor A domain-containing protein 1 | VWA1 | *Bos taurus* (Bovine) | 0.0013375 | 0.0116 | 2.9 | 1.29 | -1.61 |
| A0A452DJ03.A0A452DJ03_BOVIN | von Willebrand factor A domain-containing protein 1 | VWA1 | *Bos taurus* (Bovine) | 0.0013375 | 0.0116 | 2.9 | 1.29 | -1.61 |
| A6QLN9.VWA1_BOVIN | von Willebrand factor A domain-containing protein 1 | VWA1 | *Bos taurus* (Bovine) | 0.0013375 | 0.0116 | 2.9 | 1.29 | -1.61 |
| XP_024830811.1 | collagen alpha-1(XVIII) chain isoform X2 | COL18A1 | *Bos taurus* (Bovine) | 0.0005291 | 0.00366 | 2.85 | 1.42 | -1.42 |
| NP_001076857.1 | collagen alpha-1(XVIII) chain | COL18A1 | *Bos taurus* (Bovine) | 0.0005291 | 0.00366 | 2.85 | 1.42 | -1.42 |
| XP_024830782.1 | collagen alpha-1(XVIII) chain isoform X1 | COL18A1 | *Bos taurus* (Bovine) | 0.0005291 | 0.00366 | 2.85 | 1.42 | -1.42 |
| A4FV05.A4FV05_BOVIN | collagen alpha-1(XVIII) chain | COL18A1 | *Bos taurus* (Bovine) | 0.0005291 | 0.00366 | 2.85 | 1.42 | -1.42 |
| XP_024830844.1 | collagen alpha-1(XVIII) chain | COL18A1 | *Bos taurus* (Bovine) | 0.0005291 | 0.00366 | 2.85 | 1.42 | -1.42 |
| XP_024830882.1 | collagen alpha-1(XVIII) chain isoform X4 | COL18A1 | *Bos taurus* (Bovine) | 0.0005291 | 0.00366 | 2.85 | 1.42 | -1.42 |
| F1N6W9.F1N6W9_BOVIN | collagen alpha-1(XVIII) chain | COL18A1 | *Bos taurus* (Bovine) | 0.0005291 | 0.00366 | 2.85 | 1.42 | -1.42 |
| XP_005202100.1 | ICOS ligand isoform X2 | ICOSLG | *Bos taurus* (Bovine) | 0.000429 | 0.000325 | 2.8 | 1.2 | -1.6 |
| A0A452DIL6.A0A452DIL6_BOVIN | Glypican-1 | GPC1 | *Bos taurus* (Bovine) | 8.26E-05 | 7.34E-05 | 2.8 | 1.4 | -1.4 |
| G3X745.GPC1_BOVIN | Glypican-1 | GPC1 | *Bos taurus* (Bovine) | 8.26E-05 | 7.34E-05 | 2.8 | 1.4 | -1.4 |
| XP_010802238.2 | Glypican-1 | GPC1 | *Bos taurus* (Bovine) | 8.26E-05 | 7.34E-05 | 2.8 | 1.4 | -1.4 |
| A0A3Q1M1A9.A0A3Q1M1A9_BOVIN | MSLN protein (Mesothelin) | MSLN | *Bos taurus* (Bovine) | 0.0002846 | 0.00119 | 2.55 | 1.27 | -1.27 |
| NP_001093844.1 | MSLN protein (Mesothelin) | MSLN | *Bos taurus* (Bovine) | 0.0002846 | 0.00119 | 2.55 | 1.27 | -1.27 |
| A6QP39.A6QP39_BOVIN | MSLN protein (Mesothelin) | MSLN | *Bos taurus* (Bovine) | 0.0002846 | 0.00119 | 2.55 | 1.27 | -1.27 |
| A0A3Q1MDA5.A0A3Q1MDA5_BOVIN | Zinc finger protein 395 | ZNF395 | *Bos taurus* (Bovine) | 0.0002846 | 0.00119 | 2.55 | 1.27 | -1.27 |
| M5FJX0.M5FJX0_BOVIN | Zinc finger protein 395 | ZNF395 | *Bos taurus* (Bovine) | 0.0002846 | 0.00119 | 2.55 | 1.27 | -1.27 |
| NP_001015613.1 | Creatine kinase B-type | CKB | *Bos taurus* (Bovine) | 0.0001948 | 0.00025 | 2.54 | 1.13 | -1.41 |
| Q5EA61.KCRB_BOVIN | Creatine kinase B-type | CKB | *Bos taurus* (Bovine) | 0.0001948 | 0.00025 | 2.54 | 1.13 | -1.41 |
| E1BJ08.E1BJ08_BOVIN | Glutathione S-transferase omega | GSTO | *Bos taurus* (Bovine) | 0.0033042 | 0.0351 | 2.47 | 0.928 | -1.55 |
| XP_005225748.2 | Glutathione S-transferase omega-1 isoform X1 | GSTO | *Bos taurus* (Bovine) | 0.0033042 | 0.0351 | 2.47 | 0.928 | -1.55 |
| G5E6Q7.G5E6Q7_BOVIN | HECT domain E3 ubiquitin protein ligase 4 | HECTD4 | *Bos taurus* (Bovine) | 0.0015752 | 0.0109 | 2.45 | 0.917 | -1.53 |
| A0A3Q1MH11.A0A3Q1MH11_BOVIN | HECT domain E3 ubiquitin protein ligase 4 | HECTD4 | *Bos taurus* (Bovine) | 0.0015752 | 0.0109 | 2.45 | 0.917 | -1.53 |
| F1MY51.F1MY51_BOVIN | Peroxisome proliferator-activated receptor gamma coactivator-related protein 1 | PPRC1 | *Bos taurus* (Bovine) | 0.0015752 | 0.0109 | 2.45 | 0.917 | -1.53 |
| G3N244.G3N244_BOVIN | T-box transcription factor 22 | TBX22 | *Bos taurus* (Bovine) | 0.0015752 | 0.0109 | 2.45 | 0.917 | -1.53 |
| XP_010812363.1 | Probable E3 ubiquitin-protein ligase HECTD4 isoform X5 | HECTD4 | *Bos taurus* (Bovine) | 0.0015752 | 0.0109 | 2.45 | 0.917 | -1.53 |
| XP_015331014.1 | Probable E3 ubiquitin-protein ligase HECTD4 isoform X6 | HECTD4 | *Bos taurus* (Bovine) | 0.0015752 | 0.0109 | 2.45 | 0.917 | -1.53 |
| E1BM44.E1BM44_BOVIN | Probable E3 ubiquitin-protein ligase HECTD4 isoform X6 | HECTD4 | *Bos taurus* (Bovine) | 0.0015752 | 0.0109 | 2.45 | 0.917 | -1.53 |
| XP_010812361.2 | Probable E3 ubiquitin-protein ligase HECTD4 isoform X4 | HECTD4 | *Bos taurus* (Bovine) | 0.0015752 | 0.0109 | 2.45 | 0.917 | -1.53 |
| XP_024833531.1 | Probable E3 ubiquitin-protein ligase HECTD4 isoform X1 | HECTD4 | *Bos taurus* (Bovine) | 0.0015752 | 0.0109 | 2.45 | 0.917 | -1.53 |
| A0A2N8U234.A0A2N8U234_MYCBV | Aspartate--tRNA ligase | aspS | *Mycoplasma bovis* | 0.0011261 | 0.0119 | 2.44 | 1.22 | -1.22 |
| NP_001099108.1 | PRPF8 protein | PRPF8 | *Bos taurus* (Bovine) | 0.0006734 | 0.00212 | 2.39 | 0.896 | -1.49 |
| XP_024835577.1 | PRPF8 protein | PRPF8 | *Bos taurus* (Bovine) | 0.0006734 | 0.00212 | 2.39 | 0.896 | -1.49 |
| A7Z025.A7Z025_BOVIN | PRPF8 protein | PRPF8 | *Bos taurus* (Bovine) | 0.0006734 | 0.00212 | 2.39 | 0.896 | -1.49 |
| XP_005220137.1 | PRPF8 protein | PRPF8 | *Bos taurus* (Bovine) | 0.0006734 | 0.00212 | 2.39 | 0.896 | -1.49 |
| A0A3Q1LSG3.A0A3Q1LSG3_BOVIN | non-specific serine/threonine protein kinase | CSNK2A1 | *Bos taurus* (Bovine) | 0.0002887 | 0.000682 | 2.34 | 1.04 | -1.3 |
| P68399.CSK21_BOVIN | Casein kinase II subunit alpha | CSNK2A2 | *Bos taurus* (Bovine) | 0.0002887 | 0.000682 | 2.34 | 1.04 | -1.3 |
| NP_777060.2 | Casein kinase II subunit alpha | CSNK2A1 | *Bos taurus* (Bovine) | 0.0002887 | 0.000682 | 2.34 | 1.04 | -1.3 |
| NP_001094618.1 | BCL2 like 12 | BCL2L12 | *Bos taurus* (Bovine) | 0.0015581 | 0.00707 | 2.16 | 0.924 | -1.23 |
| A6QPJ9.A6QPJ9_BOVIN | BCL2 like 12 | BCL2L12 | *Bos taurus* (Bovine) | 0.0015581 | 0.00707 | 2.16 | 0.924 | -1.23 |
| A0A3Q1LRD9.A0A3Q1LRD9_BOVIN | BCL2 like 12 | BCL2L12 | *Bos taurus* (Bovine) | 0.0015581 | 0.00707 | 2.16 | 0.924 | -1.23 |
| XP_010813368.1 | BCL2 like 12 | BCL2L12 | *Bos taurus* (Bovine) | 0.0015581 | 0.00707 | 2.16 | 0.924 | -1.23 |
| XP_005219365.1 | BCL2 like 12 | BCL2L12 | *Bos taurus* (Bovine) | 0.0015581 | 0.00707 | 2.16 | 0.924 | -1.23 |
| Q9GMB8.SYSC_BOVIN | Serine--tRNA ligase, cytoplasmic | SARS1 | *Bos taurus* (Bovine) | 0.0022379 | 0.0325 | 1.93 | 0.965 | -0.965 |
| NP_776600.1 | Serine--tRNA ligase, cytoplasmic | SARS1 | *Bos taurus* (Bovine) | 0.0022379 | 0.0325 | 1.93 | 0.965 | -0.965 |
| Q5E9E2.MYL9_BOVIN | Myosin regulatory light polypeptide 9 | MYL9 | *Bos taurus* (Bovine) | 0.0011941 | 0.01 | 1.89 | 0.838 | -1.05 |
| XP_005224258.1 | Myosin regulatory light polypeptide 9 | MYL9 | *Bos taurus* (Bovine) | 0.0011941 | 0.01 | 1.89 | 0.838 | -1.05 |
| XP_005224259.1 | Myosin regulatory light polypeptide 9 | MYL9 | *Bos taurus* (Bovine) | 0.0011941 | 0.01 | 1.89 | 0.838 | -1.05 |
| NP_001015640.1 | Myosin regulatory light polypeptide 9 | MYL9 | *Bos taurus* (Bovine) | 0.0011941 | 0.01 | 1.89 | 0.838 | -1.05 |
| A4IF97.ML12B_BOVIN | Myosin regulatory light polypeptide 9 | MYL9 | *Bos taurus* (Bovine) | 0.0011941 | 0.01 | 1.89 | 0.838 | -1.05 |
| NP_001077233.1 | Myosin regulatory light polypeptide 9 | MYL9 | *Bos taurus* (Bovine) | 0.0011941 | 0.01 | 1.89 | 0.838 | -1.05 |
| NP_001231069.1 | Golgi apparatus protein 1 | GLG1 | *Bos taurus* (Bovine) | 0.0028707 | 0.0207 | 1.86 | 0.799 | -1.07 |
| G3N2K4.G3N2K4_BOVIN | Golgi apparatus protein 1 | GLG1 | *Bos taurus* (Bovine) | 0.0028707 | 0.0207 | 1.86 | 0.799 | -1.07 |
| E1BDY3.E1BDY3_BOVIN | Golgi apparatus protein 1 | GLG1 | *Bos taurus* (Bovine) | 0.0028707 | 0.0207 | 1.86 | 0.799 | -1.07 |
| A0A3Q1LL67.A0A3Q1LL67_BOVIN | Golgi apparatus protein 1 | GLG1 | *Bos taurus* (Bovine) | 0.0028707 | 0.0207 | 1.86 | 0.799 | -1.07 |
| E1BNE7.E1BNE7_BOVIN | Caveolae associated protein 1 | CAVIN1 | *Bos taurus* (Bovine) | 0.0022513 | 0.0264 | 1.84 | 0.818 | -1.02 |
| NP_001104573.1 | Caveolae associated protein 1 | CAVIN1 | *Bos taurus* (Bovine) | 0.0022513 | 0.0264 | 1.84 | 0.818 | -1.02 |
| F6QQB6.F6QQB6_BOVIN | FERM domain-containing protein 8 | FRMD8 | *Bos taurus* (Bovine) | 0.0032157 | 0.0251 | 1.78 | 0.765 | -1.02 |
| XP_005227137.2 | FERM domain-containing protein 8 | FRMD8 | *Bos taurus* (Bovine) | 0.0032157 | 0.0251 | 1.78 | 0.765 | -1.02 |
| A5PK03.A5PK03_BOVIN | FERM domain-containing protein 8 | FRMD8 | *Bos taurus* (Bovine) | 0.0032157 | 0.0251 | 1.78 | 0.765 | -1.02 |
| NP_001092455.1 | FERM domain-containing protein 8 | FRMD8 | *Bos taurus* (Bovine) | 0.0032157 | 0.0251 | 1.78 | 0.765 | -1.02 |
| E1BPY6.E1BPY6_BOVIN | Vacuolar protein sorting-associated protein 8 homolog | VPS8 | *Bos taurus* (Bovine) | 0.0006106 | 0.00478 | 1.7 | 0.848 | -0.848 |
| XP_024851058.1 | Vacuolar protein sorting-associated protein 8 homolog | VPS8 | *Bos taurus* (Bovine) | 0.0006106 | 0.00478 | 1.7 | 0.848 | -0.848 |
| XP_024851049.1 | Vacuolar protein sorting-associated protein 8 homolog | VPS8 | *Bos taurus* (Bovine) | 0.0006106 | 0.00478 | 1.7 | 0.848 | -0.848 |
| A0A2N8U253.A0A2N8U253_MYCBV | Multifunctional lipase Mycoplasma immunogenic lipase A | milA | *Mycoplasma bovis* | 0.0023836 | 0.0351 | 1.56 | 0.78 | -0.78 |
| NP_001028780.1 | Macrophage migration inhibitory factor | MIF | *Bos taurus* (Bovine) | 0.0026976 | 0.0404 | 1.52 | 0.759 | -0.759 |
| A0A0F7RPX0.A0A0F7RPX0_BOVIN | Macrophage migration inhibitory factor | MIF | *Bos taurus* (Bovine) | 0.0026976 | 0.0404 | 1.52 | 0.759 | -0.759 |
| P80177.MIF_BOVIN | Macrophage migration inhibitory factor | MIF | *Bos taurus* (Bovine) | 0.0026976 | 0.0404 | 1.52 | 0.759 | -0.759 |
| XP_024833856.1 | Adhesion G-protein coupled receptor G1 | ADGRG1 | *Bos taurus* (Bovine) | 0.0035779 | 0.0389 | -1.87 | -0.701 | 1.17 |
| G3N239.G3N239_BOVIN | Adhesion G-protein coupled receptor G1 | ADGRG1 | *Bos taurus* (Bovine) | 0.0035779 | 0.0389 | -1.87 | -0.701 | 1.17 |
| XP_024833853.1 | Adhesion G-protein coupled receptor G1 | ADGRG1 | *Bos taurus* (Bovine) | 0.0035779 | 0.0389 | -1.87 | -0.701 | 1.17 |
| A0A3Q1M967.A0A3Q1M967_BOVIN | Adhesion G-protein coupled receptor G1 | ADGRG1 | *Bos taurus* (Bovine) | 0.0035779 | 0.0389 | -1.87 | -0.701 | 1.17 |
| A0A3Q1LXM5.A0A3Q1LXM5_BOVIN | Adhesion G-protein coupled receptor G1 | ADGRG1 | *Bos taurus* (Bovine) | 0.0035779 | 0.0389 | -1.87 | -0.701 | 1.17 |
| NP_001077125.1 | Adhesion G-protein coupled receptor G1 | GPR56 | *Bos taurus* (Bovine) | 0.0035779 | 0.0389 | -1.87 | -0.701 | 1.17 |
| A4IF70.A4IF70_BOVIN | Adhesion G-protein coupled receptor G1 | GPR56 | *Bos taurus* (Bovine) | 0.0035779 | 0.0389 | -1.87 | -0.701 | 1.17 |
| F1MKM4.F1MKM4_BOVIN | Adhesion G-protein coupled receptor G1 | ADGRG1 | *Bos taurus* (Bovine) | 0.0035779 | 0.0389 | -1.87 | -0.701 | 1.17 |
| XP_024833855.1 | Adhesion G-protein coupled receptor G1 | ADGRG1 | *Bos taurus* (Bovine) | 0.0035779 | 0.0389 | -1.87 | -0.701 | 1.17 |
| XP_024833857.1 | Adhesion G-protein coupled receptor G1 | ADGRG1 | *Bos taurus* (Bovine) | 0.0035779 | 0.0389 | -1.87 | -0.701 | 1.17 |
| XP_024833852.1 | Adhesion G-protein coupled receptor G1 | ADGRG1 | *Bos taurus* (Bovine) | 0.0035779 | 0.0389 | -1.87 | -0.701 | 1.17 |
| XP_024833854.1 | Adhesion G-protein coupled receptor G1 | ADGRG1 | *Bos taurus* (Bovine) | 0.0035779 | 0.0389 | -1.87 | -0.701 | 1.17 |
| XP_010812603.1 | Lysine--tRNA ligase | KARS1 | *Bos taurus* (Bovine) | 0.0027997 | 0.0278 | -2.01 | -0.752 | 1.25 |
| F1MMK8.F1MMK8_BOVIN | Lysine--tRNA ligase | KARS1 | *Bos taurus* (Bovine) | 0.0027997 | 0.0278 | -2.01 | -0.752 | 1.25 |
| NP_001029528.1 | Lysine--tRNA ligase | KARS1 | *Bos taurus* (Bovine) | 0.0027997 | 0.0278 | -2.01 | -0.752 | 1.25 |
| Q3T0N2.Q3T0N2_BOVIN | Lysine--tRNA ligase | KARS1 | *Bos taurus* (Bovine) | 0.0027997 | 0.0278 | -2.01 | -0.752 | 1.25 |
| NP_001095677.1 | Exocyst complex component Sec8 | EXOC4 | *Bos taurus* (Bovine) | 0.0030605 | 0.0316 | -2.11 | -1.05 | 1.05 |
| XP_024846557.1 | Exocyst complex component Sec8 | EXOC4 | *Bos taurus* (Bovine) | 0.0030605 | 0.0316 | -2.11 | -1.05 | 1.05 |
| A6QLD1.A6QLD1_BOVIN | Exocyst complex component Sec8 | EXOC4 | *Bos taurus* (Bovine) | 0.0030605 | 0.0316 | -2.11 | -1.05 | 1.05 |
| Q5EA01.B4GA1_BOVIN | Beta-1,4-glucuronyltransferase 1 | B4GAT1 | *Bos taurus* (Bovine) | 0.0016823 | 0.0119 | -2.11 | -0.792 | 1.32 |
| NP_001094547.1 | 26S proteasome non-ATPase regulatory subunit 1 | PSMD1 | *Bos taurus* (Bovine) | 0.0012159 | 0.00723 | -2.26 | -0.849 | 1.41 |
| XP_010800708.1 | 26S proteasome non-ATPase regulatory subunit 1 | PSMD1 | *Bos taurus* (Bovine) | 0.0012159 | 0.00723 | -2.26 | -0.849 | 1.41 |
| A7MBA2.A7MBA2_BOVIN | 26S proteasome non-ATPase regulatory subunit 1 | PSMD1 | *Bos taurus* (Bovine) | 0.0012159 | 0.00723 | -2.26 | -0.849 | 1.41 |
| A0A2N8U2Q5.A0A2N8U2Q5_MYCBV | DUF3137 domain-containing protein | HYD69_01635 | *Mycoplasma bovis* | 0.0017935 | 0.0243 | -2.4 | -1.2 | 1.2 |
| A0A2N8U1V9.A0A2N8U1V9_MYCBV | CvpA family protein | MBOVJF4278_00174 | *Mycoplasma bovis* | 0.0041139 | 0.0364 | -2.51 | -1.43 | 1.07 |
| XP_015327629.1 | Adiponectin, C1Q and collagen domain containing | ADIPOQ | *Bos taurus* (Bovine) | 0.0032217 | 0.0417 | -2.66 | -1.48 | 1.18 |
| XP_024851000.1 | Adiponectin | ADIPOQ | *Bos taurus* (Bovine) | 0.0032217 | 0.0417 | -2.66 | -1.48 | 1.18 |
| A0A3B0J3V0.A0A3B0J3V0_BOVIN | Adiponectin D | ADID | *Bos taurus* (Bovine) | 0.0032217 | 0.0417 | -2.66 | -1.48 | 1.18 |
| Q3Y5Z3.ADIPO_BOVIN | TBC1 domain family member 30 | TBC1D30 | *Bos taurus* (Bovine) | 0.0032217 | 0.0417 | -2.66 | -1.48 | 1.18 |
| A0A3Q1M564.A0A3Q1M564_BOVIN | TBC1 domain family member 30 | TBC1D30 | *Bos taurus* (Bovine) | 0.0032217 | 0.0417 | -2.66 | -1.48 | 1.18 |
| XP_024851001.1 | Adiponectin, C1Q and collagen domain containing | ADIPOQ | *Bos taurus* (Bovine) | 0.0032217 | 0.0417 | -2.66 | -1.48 | 1.18 |
| XP_024853571.1 | charged multivesicular body protein 4a isoform X1 | CHMP4A | *Bos taurus* (Bovine) | 0.0031809 | 0.0495 | -2.7 | -1.35 | 1.35 |
| NP_001121976.1 | Charged multivesicular body protein 4a | CHMP4A | *Bos taurus* (Bovine) | 0.0031809 | 0.0495 | -2.7 | -1.35 | 1.35 |
| A2VDY3.CHM4A_BOVIN | Charged multivesicular body protein 4a | CHMP4A | *Bos taurus* (Bovine) | 0.0031809 | 0.0495 | -2.7 | -1.35 | 1.35 |
| A0A1B0Z6M0.A0A1B0Z6M0_MYCBV | Dihydrolipoyl dehydrogenase | pdhD | *Mycoplasma bovis* | 0.0003722 | 0.0019 | -2.78 | -1.39 | 1.39 |
| Q4U0X8.Q4U0X8_MYCBV | Charged multivesicular body protein 4a isoform X1 | CHMP4A | *Bos taurus* (Bovine) | 0.002642 | 0.0395 | -2.78 | -1.39 | 1.39 |
| A0A2N8U2G5.A0A2N8U2G5_MYCBV | Peptide methionine sulfoxide reductase MsrA | msrB | *Mycoplasma bovis* | 0.007751 | 0.0479 | -2.8 | -1.68 | 1.12 |
| A0A2N8U224.A0A2N8U224_MYCBV | Putative NADP-dependent isopropanol dehydrogenase or Zinc-dependent alcohol dehydrogenase family protein | HYD69_02450 | *Mycoplasma bovis* | 0.004784 | 0.0449 | -2.94 | -1.26 | 1.68 |
| A0A2N8U1Y8.A0A2N8U1Y8_MYCBV | Isoleucine--tRNA ligase | ileS | *Mycoplasma bovis* | 0.0004062 | 0.000737 | -2.96 | -1.48 | 1.48 |
| A0A2N8U230.A0A2N8U230_MYCBV | Small ribosomal subunit protein uS2 | rpsB | *Mycoplasma bovis* | 0.0043603 | 0.0394 | -3.06 | -1.31 | 1.75 |
| XP_024855992.1 | breast cancer type 2 susceptibility protein isoform X3 | BRCA2 | *Bos taurus* (Bovine) | 8.57E-05 | 1.06E-06 | -3.1 | -1.33 | 1.77 |
| E1B8X8.E1B8X8_BOVIN | BRCA2 DNA repair associated | BRCA2 | *Bos taurus* (Bovine) | 8.57E-05 | 1.06E-06 | -3.1 | -1.33 | 1.77 |
| XP_024855991.1 | breast cancer type 2 susceptibility protein isoform X2 | BRCA2 | *Bos taurus* (Bovine) | 8.57E-05 | 1.06E-06 | -3.1 | -1.33 | 1.77 |
| XP_024855990.1 | breast cancer type 2 susceptibility protein homolog isoform 1 | BRCA2 | *Bos taurus* (Bovine) | 8.57E-05 | 1.06E-06 | -3.1 | -1.33 | 1.77 |
| A0A2N8U1F3.A0A2N8U1F3_MYCBV | Membrane protein insertase YidC | yidC | *Mycoplasma bovis* | 0.0001622 | 0.000166 | -3.16 | -1.41 | 1.76 |
| F1MGR1.F1MGR1_BOVIN | Sodium/nucleoside cotransporter | SLC28A3 | *Bos taurus* (Bovine) | 0.0018789 | 0.00982 | -3.2 | -1.83 | 1.37 |
| NP_001179096.1 | Sodium/nucleoside cotransporter | SLC28A3 | *Bos taurus* (Bovine) | 0.0018789 | 0.00982 | -3.2 | -1.83 | 1.37 |
| XP_024851313.1 | Sodium/nucleoside cotransporter | SLC28A3 | *Bos taurus* (Bovine) | 0.0018789 | 0.00982 | -3.2 | -1.83 | 1.37 |
| XP_015328102.1 | Sodium/nucleoside cotransporter | SLC28A3 | *Bos taurus* (Bovine) | 0.0018789 | 0.00982 | -3.2 | -1.83 | 1.37 |
| A0A2N8U2Q7.A0A2N8U2Q7_MYCBV | Phosphopentomutase | deoB | *Mycoplasma bovis* | 0.0012008 | 0.0132 | -3.25 | -1.63 | 1.63 |
| E1BAQ3.E1BAQ3_BOVIN | Mucin 13, cell surface associated | MUC13 | *Bos taurus* (Bovine) | 0.001837 | 0.0251 | -3.46 | -1.73 | 1.73 |
| XP_002684842.4 | Mucin 13, cell surface associated | MUC13 | *Bos taurus* (Bovine) | 0.001837 | 0.0251 | -3.46 | -1.73 | 1.73 |
| A0A2N8U256.A0A2N8U256_MYCBV | alcohol dehydrogenase | adh | *Mycoplasma bovis* | 0.0002588 | 0.000986 | -3.67 | -1.84 | 1.84 |
| A0A2N8U1G9.A0A2N8U1G9_MYCBV | Phosphatidylglycerol--prolipoprotein diacylglyceryl transferase | lgt | *Mycoplasma bovis* | 0.0028835 | 0.0128 | -3.86 | -3.22 | 0.643 |
| A0A2N8U2T2.A0A2N8U2T2_MYCBV | P80 family lipoprotein or  Putative lipoprotein MPN_284 | HYD69_01350 | *Mycoplasma bovis* | 0.000131 | 0.000205 | -4.07 | -2.04 | 2.04 |
| A0A193CK90.A0A193CK90_MYCBV | 2-oxoisovalerate dehydrogenase subunit alpha or Branched-chain alpha-keto acid dehydrogenase E1 component alpha chain | BC94_0086 | *Mycoplasma bovis* | 1.26E-05 | 1.89E-07 | -4.24 | -2.12 | 2.12 |
| A0A2N8U2L3.A0A2N8U2L3_MYCBV | ATP synthase subunit beta 2 | MBOVJF4278_00494 | *Mycoplasma bovis* | 0.0014929 | 0.0101 | -4.28 | -2.68 | 1.61 |
| A0A193CK94.A0A193CK94_MYCBV | Pyruvate dehydrogenase E1 component subunit beta | PDHB | *Mycoplasma bovis* | 9.30E-05 | 9.88E-05 | -4.33 | -2.16 | 2.16 |
| A0A2N8U273.A0A2N8U273_MYCBV | ABC transporter substrate-binding protein or High affinity transport system protein p37 | SBP | *Mycoplasma bovis* | 0.0012316 | 0.00739 | -4.39 | -2.74 | 1.65 |
| A0A2N8U3J8.A0A2N8U3J8_MYCBV | Probable cytosol aminopeptidase or Leucine aminopeptidase | BC94_0699 | *Mycoplasma bovis* | 0.0001095 | 6.30E-05 | -4.61 | -2.56 | 2.05 |
| A0A2N8U2Q4.A0A2N8U2Q4_MYCBV | L-lactate dehydrogenase | HYD69_01415 | *Mycoplasma bovis* | 1.93E-05 | 8.10E-07 | -4.61 | -2.31 | 2.31 |
| A0A2N8U3I7.A0A2N8U3I7_MYCBV | Variable surface lipoprotein | MBOVJF4278_00818 | *Mycoplasma bovis* | 3.04E-06 | 4.38E-10 | -4.7 | -2.35 | 2.35 |
| A0A2N8U1I5.A0A2N8U1I5_MYCBV | Dihydrolipoamide acetyltransferase component of pyruvate dehydrogenase complex | BC94_0088 | *Mycoplasma bovis* | 1.82E-05 | 6.84E-07 | -4.7 | -2.35 | 2.35 |
| Q3SZB8.H3CL_BOVIN | Histone H3.3C-like | H3.3 | *Bos taurus* (Bovine) | 0.0074282 | 0.0262 | -4.92 | -2.46 | 2.46 |
| NP_001069659.1 | Histone H3.3C-like | H3.3 | *Bos taurus* (Bovine) | 0.0074282 | 0.0262 | -4.92 | -2.46 | 2.46 |
| A0A2N8U1H9.A0A2N8U1H9_MYCBV | Lipoprotein | HYD69_00530 | *Mycoplasma bovis* | 7.83E-05 | 6.48E-05 | -5.33 | -2.66 | 2.66 |
| A0A2N8U2M1.A0A2N8U2M1_MYCBV | Elongation factor Tu | tuf | *Mycoplasma bovis* | 7.98E-07 | 2.93E-13 | -5.66 | -2.83 | 2.83 |
